# Pyrrole without Life: Reaction of Aminomethylene with the Propargyl Radical

**DOI:** 10.1021/acs.jpclett.5c03948

**Published:** 2026-02-04

**Authors:** Rory McClish, Domenik Schleier, Jerry Kamer, Tina Kasper, Patrick Hemberger, Andras Bodi, Jordy Bouwman

**Affiliations:** † Department of Chemistry, 1877 University of Colorado, Boulder, Colorado 80309, United States; ‡ Laboratory for Atmospheric and Space Physics, University of Colorado, Boulder, Colorado 80303, United States; ¶ Institut für Physik und Astronomie, Technische Universität Berlin, Hardenbergstr. 36, 10623 Berlin, Germany; § Laboratory for Astrophysics Leiden Observatory, 98812 Leiden University, NL 2300 RA Leiden, The Netherlands; ∥ Lehrstuhl Technische Thermodynamik, Fakultät für Maschinenbau, Universität Paderborn, Warburger Str. 100, 33098 Paderborn, Germany; ⊥ Laboratory for Synchrotron Radiation and Femtochemistry, Paul Scherrer Insitute (PSI), 5232 Villigen, Switzerland; # Institute for Modeling Plasma, Atmospheres and Cosmic Dust (IMPACT), NASA/SSERVI, Boulder, Colorado 80309, United States

## Abstract

Carbenes are reactive species found across gas-phase environments, from combustion to planetary atmospheres and interstellar space. Their reactions with radicals represent a compelling path to increasing chemical complexity, in which the formation of the first aromatic ring is a foundational step. To date, no selective gas-phase bottom-up route to the smallest nitrogen-bearing aromatic ring, pyrrole, is known. We investigated the reaction of the simplest aminocarbene, aminomethylene, with the prototypical resonance stabilized propargyl radical. Photoelectron photoion coincidence spectroscopy and semiautomated electronic structure calculations reveal a barrierless, addition–elimination mechanism producing pyrrole + H. The reaction path depends on the orientation of propargyl during the association, in which the allenyl resonance form (H_2_C=C=CH^•^) of propargyl leads to pyrrole formation. This selective pathway highlights the promise of radical chemistry to fill important gaps in chemical reaction networks.

## Graphical abstract

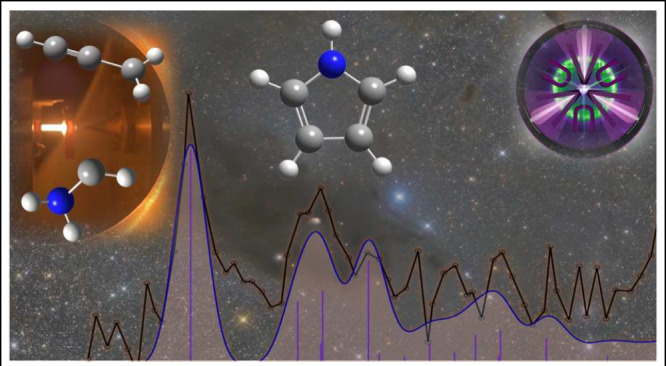

Small carbenes, composed of up to five heavy atoms, are present in gas-phase environments spanning combustion, planetary atmospheres, and the interstellar medium (ISM).^1–5^ Early observations detected the simplest carbene, methylene,^6^ CH_2_, as well as cyclopropenylidene, *c*-C_3_H_2_, in the ISM.^7^ The diverse inventory of interstellar carbenes grew as systematic detections were made by radio astronomy in conjunction with laboratory studies.^8^

Further laboratory characterization efforts^9–13^ have established that the molecular geometry and reactivity of a carbene is influenced by its spin multiplicity, and substituent groups can modify the energetic ordering of electronic states. The amine group is a lone-pair donating substituent; accordingly, substitution of a hydrogen in methylene (X̃ ^3^B_1_) with NH_2_ stabilizes the smallest aminocarbene, aminomethylene (HCNH_2_, **AM**), in its closed-shell electronic ground state, ^1^A′. Recently, Eckhardt and Schreiner^14^ synthesized **AM** in the gas phase through high-vacuum flash pyrolysis of cyclopropylamine (**CPA**, see Scheme 1). The **CPA** pyrolysate was trapped in a solid argon matrix and **AM** characterized using infrared and UV/vis spectroscopy.

**Scheme 1:**
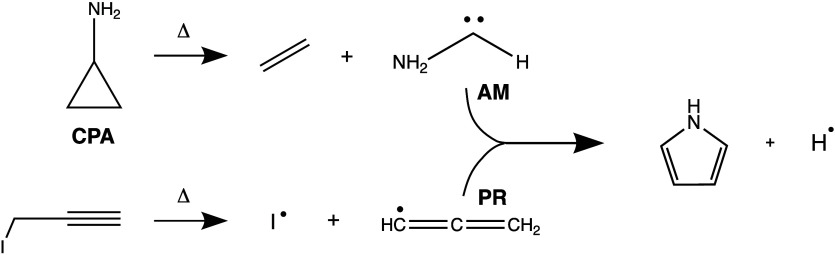
Pyrolysis Scheme to Generate the Reactants Aminomethylene (**AM**) and Propargyl Radical (**PR**) *in Situ* from Cyclopropylamine (**CPA**) and Propargyl Iodide, Respectively, and Their Association Reaction to Form Pyrrole

The enhanced stability of **AM** contextualizes its proposed role in prebiotic chemical networks as an intermediate in the formation of sugars and amino acids.^14,15^ Beyond these biomolecules, small carbenes like **AM**, or other carbenes for which **AM** is a model, may contribute more broadly to the bottom-up development of chemical complexity, including the formation of (hetero)­cyclic aromatic molecules. In both the ISM and planetary atmospheres, the origin and evolution of N-bearing heterocyclic molecules remains a confounding question.^16–19^ Nitrogen has so far only been found in cyano-group moieties of interstellar polycyclic aromatic hydrocarbons (PAHs).^8^ This can be partially attributed to detection bias in radioastronomy toward cyano-substituted cyclic aromatics due to the large permanent dipole moment imparted by the nitrile substituent. N-Heterocycle PAHs, where the heteroatom is part of the cyclic backbone, have been proposed as possible carriers of the 6.2 μm aromatic infrared bands,^20^ and a machine learning model predicts the presence of heterocyclic molecules inside the cold molecular cloud TMC-1.^21^ Moreover, N-heterocycles have been identified in asteroid and meteorite samples with isotopic abundances suggestive of an interstellar origin, including specifically cold molecular clouds.^22–24^ Their presence is particularly intriguing in the context of the formation of biologically relevant molecules in space and their delivery to planetary bodies. A mechanistic understanding of N-heterocycle synthesis could help reconcile the contradiction between the expected presence and the lack of the astronomical detection of N-heterocycle PAHs.

On Saturn’s largest moon Titan, the Cassini–Huygens mission revealed a rich chemistry involving N-heterocycles, with *m*/*z* peaks at 67 and 68 consistent with pyrrole cations and protonated pyrrole.^25^ Titan serves as a proxy for early Earth; the atmosphere is dominated by nitrogen and methane, which serve as the basis for a progression from aromatic cycles to haze formation.^17–19,26^ This progression is not fully understood, and the upcoming Dragonfly mission will seek to provide new insights into Titan’s chemistry.^27,28^ Laboratory investigations and chemical kinetic models are integral to interpreting the results of observational campaigns. To date, neither the astronomical database KIDA^29^ nor UMIST^30^ contain pathways to the small N-bearing aromatic rings pyrrole or pyridine.

Incorporating nitrogen into the cyclic backbone of an aromatic molecule does not proceed in analogy to gas-phase pure-PAH growth, highlighting the need for experimentally verified reactions that are viable under the given environmental constraints.^31^ Recently, Johansen et al.^32^ developed a computational methodology to predict reactant candidates for complex molecule formation. The case studies on prototypical N-heterocycles pyrrole and pyridine formation included carbene–radical reactions. Experimental studies are necessary to ground such proposals of carbene reactivity.^33^

Resonance stabilized radicals (RSRs) make for especially attractive reactants in bimolecular chemistry with carbenes from the perspective of astrochemistry, where mechanisms relevant to cold molecular clouds must be (nearly) barrierless. The smallest RSR, propargyl (C_3_H_3_^·^, **PR**), is an important reaction candidate due to its abundance in the ISM.^34^ Moreover, **PR** has been well-characterized in the laboratory^35–37^ and is known to play a key role in initial ring formation, hydrocarbon ring growth, and eventual soot formation.^38–43^

In this work, we cogenerated **AM** and **PR** in a resistively heated silicon carbide (SiC) pyrolysis microreactor from the precursors cyclopropylamine and propargyl iodide to study their bimolecular chemistry, as described by Scheme 1. The reaction mixture was probed by double-imaging photoelectron photoion coincidence (*i* ^2^PEPICO) spectroscopy using the tunable VUV synchrotron radiation available at the Swiss Light Source, allowing for simultaneously performing photoionization time-of-flight (TOF) mass spectrometry and mass-selected threshold photoelectron spectroscopy. Semiautomated electronic structure calculations using KinBot^44,45^ guide the analysis of the potential energy surface (PES) and allow for insights into the reaction mechanism. Combining such a holistic exploration of the PES with the multiplexed experimental approach reveals a bimolecular carbene radical reaction able to produce the first aromatic N-bearing ring from two acyclic reactants.

Photoionization time-of-flight (TOF) mass spectra are presented in Figure 1. The top trace (Figure 1A) shows the pyrolysis of propargyl iodide (I–C_3_H_3_, *m*/*z* 166) seeded in argon at a temperature of 930 K. Pyrolysis of I–C_3_H_3_ results in cleavage of the C–I bond and clean formation of **PR**, observed at *m*/*z* 39 in the 9 eV spectrum in accordance with its ionization energy (IE) of 8.70 eV.^46^ The photoion mass-selected threshold photoelectron spectrum (ms-TPES, Figure S1) of *m*/*z* 39 confirms **PR** as the sole carrier of the peak. Additionally, the peak at *m*/*z* 78 provides clear evidence of the **PR** self-reaction expected to generate a suite of C_6_H_6_ isomers, several of which ionize below 9 eV.^43,47^ Under these experimental reaction conditions, the *m*/*z* 39 and *m*/*z* 78 peaks demonstrate that **PR** is generated inside the SiC microreactor in an abundance amenable to bimolecular chemistry.

**Figure 1:**
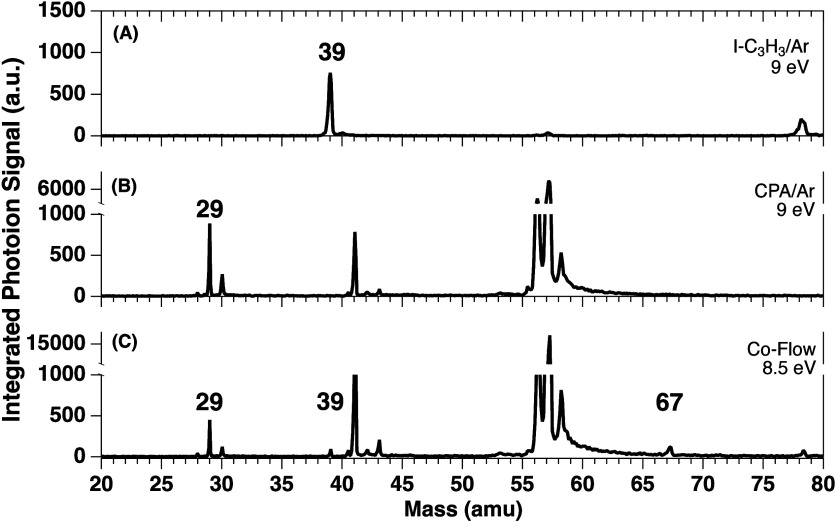
Photoionization time-of-flight mass spectra recorded for (A) propargyl iodide seeded in argon at 9 eV photon energy and 930 K pyrolysis temperature, (B) cyclopropylamine seeded in argon at 9 eV photon energy and 960 K pyrolysis temperature, and (C) cyclopropylamine and propargyl iodide seeded in argon at 8.5 eV photon energy and 900 K pyrolysis temperature.

Following the work of Eckhardt and Schreiner,^14^ we pyrolyzed a dilute mixture of **CPA** seeded in argon at 960 K (Figure 1B). The thermal decomposition product of interest, aminomethylene (**AM**, HCNH_2_), appears at *m*/*z* 29 and is distinguished from its isomer, methanimine (**MA**, H_2_C=NH), based on their IEs. The calculated IE of singlet **AM** is 8.23 eV, which is substantially lower than the IE of **MA** (9.99 eV^48^). Thus, the mass spectrum in Figure 1B collected at 9 eV indicates the successful decomposition of **CPA** to **AM** (Figure 1) inside the microreactor. Generation of the **AM** reactant is further confirmed by ms-TPES (Figure S7). Critically, the short residence time (<50 μs) in the reactor followed by a collision-free environment after the expansion quenches secondary **AM** chemistry prior to detection in the molecular beam.^49^ The coproduct to **AM** production, ethylene, does not ionize until 10.5 eV^50^ and is thus not detected in the spectrum shown in Figure 1B. For additional analysis of the pyrolysis of **CPA**, including the selection of conditions that minimize the thermal isomerization of **AM** to **MA**, the reader is referred to the Supporting Information (Figures S3–S7). In short, the signal at *m*/*z* 30 in Figure 1B is attributed to the H_2_CNH_2_ radical in combination with the ^13^C isotope contribution of **AM**. Further peaks bear witness to allyl radical (C_3_H_5_^·^, *m*/*z* 41) and vinylamine (C_2_H_3_NH_2_, *m*/*z* 43) formation. The H-loss product (*m*/*z* 56) and ^13^C isotopologue (*m*/*z* 58) bracket the main **CPA** precursor peak at *m*/*z* 57.

Figure 1C shows the mass spectrum of the coflow of both reactant precursors in the SiC reactor at 900 K. The tail of the intense *m*/*z* 57 peak toward higher times of flight is a detection artifact and diminished the dynamic range of the experiment at higher photon energies. Therefore, this spectrum was recorded at a decreased photon energy of 8.5 eV. The mass spectrum is almost the sum of the spectra in Figure 1A,B, but a new signal appears at *m*/*z* 67. Thus, this peak is due to a product formed in a bimolecular reaction between the two pyrolytic mixtures (Scheme 1).

In order to unambiguously identify the carrier of the *m*/*z* 67 peak, we recorded its ms-TPES signal (Figure 2). The first resonance in the *m*/*z* 67 ms-TPES is seen at the known adiabatic ionization potential of pyrrole previously measured at 8.20 ± 0.05 eV.^51^ Notwithstanding the suboptimal S/N, the observed origin transition at the known IE of pyrrole and the good general agreement between the band structure of the measured *m*/*z* 67 ms-TPES and the vibronic transitions calculated in the pyrrole Franck–Condon (FC) simulation, as shown in Figure 2, conclusively identify pyrrole as a reaction product.

**Figure 2:**
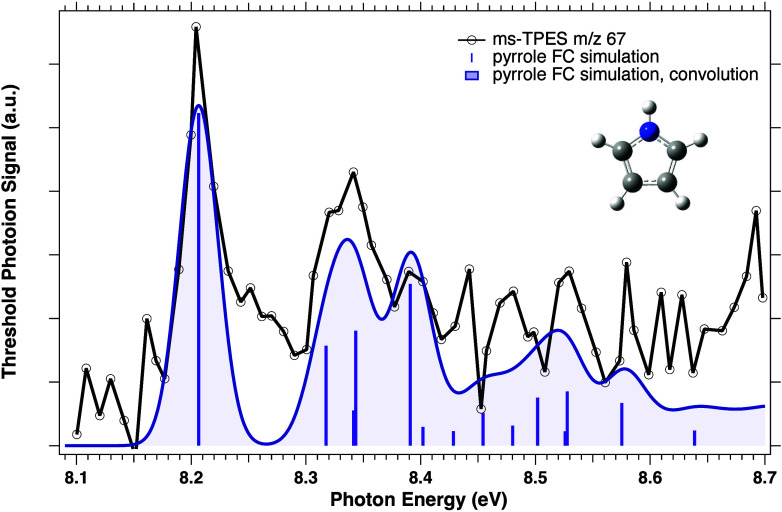
Photoion mass-selected threshold photoelectron spectrum of *m*/*z* 67 collected over the incident photon range 8.1–8.7 eV. The purple sticks represent the main vibronic transitions in the FC simulation at 300 K, while the shaded blue trace is the result of convolving all vibronic transitions with a Gaussian of 40 meV fwhm.

The mass spectrometry experiments (Figure 1) demonstrate that **PR**, a pure hydrocarbon, reacts with a **CPA** pyrolysis product to form a bimolecular reaction product detected at *m*/*z* 67. The assignment of this peak to pyrrole dictates that the coreactant to **PR** is an N-bearing species. The difference between the nominal masses of **PR** and pyrrole point to an N-bearing product of **CPA** pyrolysis with a mass of *at least* 28 amu. We thus consider both **MA** and **AM** (*m*/*z* 29) as possible coreactants to **PR** in an addition–elimination type reaction resulting in H-loss. However, investigation of the pyrolysis chemistry of **CPA** as a function of reactor temperature demonstrates negligible **MA** production under the conditions that the coflow experiment was performed (∼900 K, Figure S6). Independently, calculation of the **MA** + **PR** (C_4_H_6_N) PES finds that appreciable entrance barriers combined with high-energy, noncompetitive subsequent isomerization steps rule out the contribution of **MA** + **PR** to the production of pyrrole (Figures S8–S13 and related text). Therefore, we attribute reaction **AM** + **PR** as being responsible for the formation of pyrrole and look to insights available from the relevant portion of the C_4_H_6_N PES.

Two isomeric initial addition complexes can be formed from the association of the carbenic carbon in **AM** with either terminal of the propargyl radical, namely the “head” (the CH end of the allenyl-like H_2_C=C=CH^•^ resonance structure) or the “tail” (CH_2_ end of the ethynyl methyl-like HC≡C–CH_2_^•^ resonance structure). Therefore, the mechanism of **AM** + **PR** bifurcates *upon association* along either the **AM-headPR** or the **AM-tailPR** reaction path. Starting from the initial C_4_H_6_N adducts, the PES was explored using KinBot.^44,45^ Crucially, our use of KinBot at the DFT level explores all single reaction steps from a given input well that proceed over a transition state (TS) beneath the total energy of separated reactants **AM** and **PR**. Within a single KinBot calculation, we compared the relative energies of each TS and followed the reaction step with the lowest energy TS to the next C_4_H_6_N intermediate, from which a new KinBot calculation was initiated. This workflow enables calculating the minimum energy pathway agnostic to a specific final product. This approach is the basis for the summary PES shown in Figure 3, which thus represents the energetically most favored reaction path along both the **AM-headPR** and the **AM-tailPR** routes. The set of KinBot calculations (which include the higher lying TSs) is visualized in the Supporting Information Figures S14–S16 and S18–S20.

**Figure 3:**
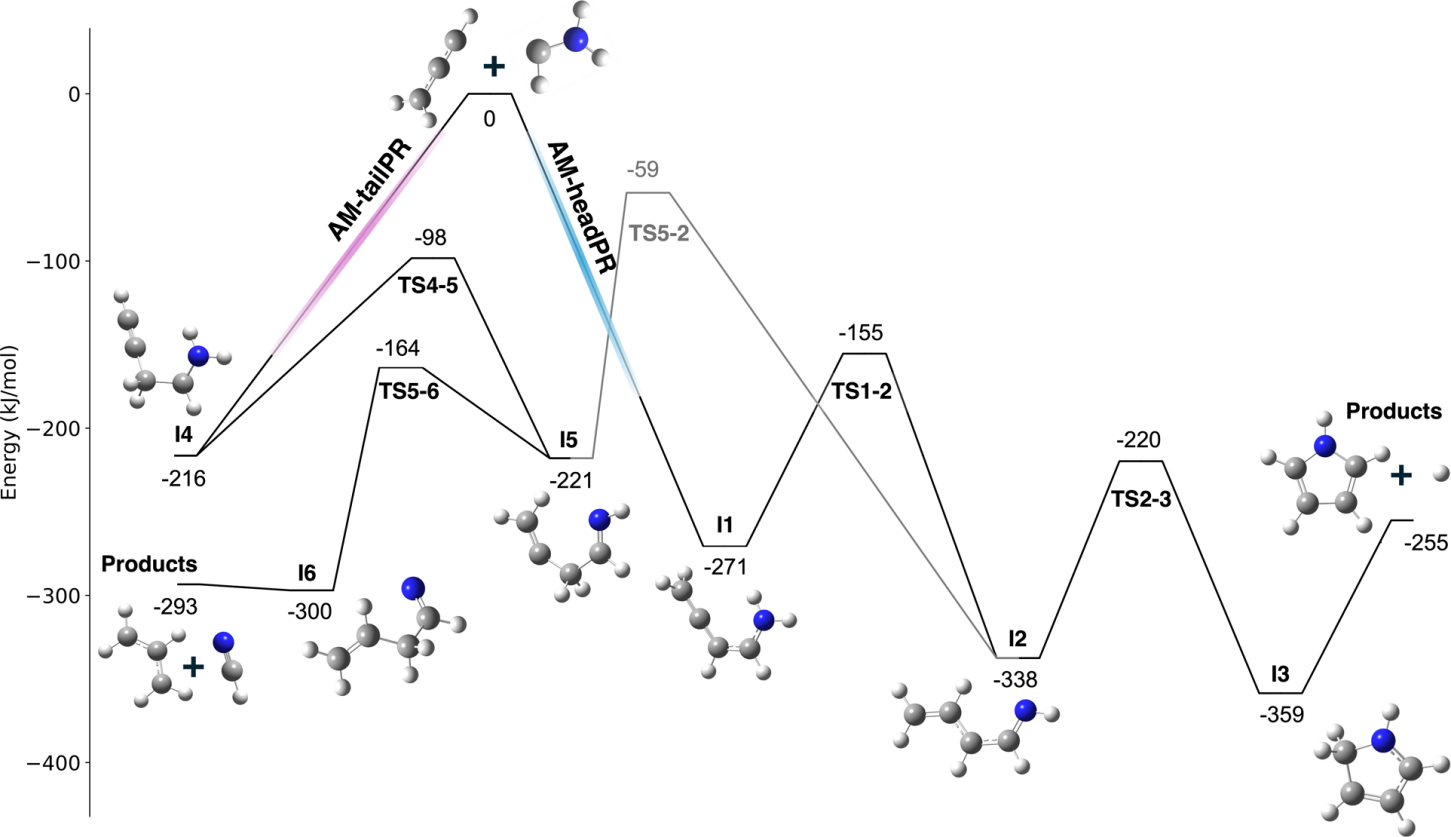
Selected portion of the HCNH_2_+C_3_H_3_ potential energy surface as explored using Kinbot. Each stationary point is calculated using CCSD­(T)/aug-cc-pVTZ//M06-2X/aug-cc-pVTZ and energies are referenced to the sum of the electronic and zero-point energies of the separated reactants.

The association of **AM** with the head of **PR** (**AM-headPR**) is exothermic by 271 kJ/mol and leads to initial intermediate **I1**. Calculations along the carbon–carbon bonding coordinate of the association reaction indicate that this step is barrierless with a slightly submerged stationary point associated with the nascent bonding coordinate (see Table S1 and accompanying discussion in the Supporting Information). Hydrogen migration from **I1** results in the lowest-lying acyclic C_4_H_6_N radical on the calculated surface, the resonance-stabilized intermediate **I2**. Ring closure to **I3** is followed by facile H-loss to the pyrrole. The entire **AM-headPR** route to pyrrole proceeds over submerged barriers, with respect to the energy of the initial reactants.

The initial addition along the **AM-tailPR** path yields intermediate **I4**, which is 55 kJ/mol higher in energy than **I1**. The reaction step from **I4** with the lowest barrier is a hydrogen transfer over **TS4–5** to **I5**. From there, **TS5–2** connects the **AM-tailPR** and **AM-headPR** addition routes but is higher in energy than **TS5–6** to **I6** by 105 kJ/mol (see Figure S19 for the full KinBot search from **I5**). We find **I6** to be slightly lower (7 kJ/mol) than the combined energy of the homolytic scission products hydrogen cyanide and the allyl radical. Direct characterization of HCN and C_3_H_5_ as bimolecular reaction products resulting from the **AM-tailPR** branch is precluded by the fact that they are known products of **CPA** pyrolysis chemistry: allyl radical is identified by ms-TPES in this work (see the mass spectrum in Figure 1B and ms-TPES in Figure S5), and while HCN was identified previously,^52^ it is not ionized in our experiment due to its high IE of 13.6 eV.^53,54^ Therefore, although the **AM-tailPR** reaction path is connected to pyrrole formation, the energetic **TS5–2** dictates that the production of pyrrole is not competitive via this route in favor of HCN + C_3_H_5_; pyrrole is predicted to be exclusively formed upon the addition of **AM** to the CH end of **PR** (**AM-headPR**).

Despite the fact that C_4_H_6_N intermediates **I1** and **I4** share the same heavy atom backbone, the initial distribution of hydrogen atoms determines the preferred hydrogen migration step through cyclic transition states. The calculations also confirm the intuition of the deep potential wells accessed upon association of a carbene and RSR, which allows for subsequent unimolecular rearrangements and an eventual H-elimination step to all take place well beneath the energy of the reactants. This is in analogy to previously established gas-phase reactions like that of *o-*benzyne + methyl and the recently investigated phenylnitrene + **PR**.^55,56^ When propargyl associates with phenylnitrene, a mechanistic bifurcation analogous to the one herein was also identified, in which the CH^•^ terminal of propargyl was found to promote ring condensation leading to quinoline.

The **AM-headPR** path thus represents a bimolecular mechanism involving neutral species that can produce pyrrole in the gas phase. The calculations suggest that the reaction will occur at low temperatures under conditions relevant to the ISM and planetary atmospheres, adding to an emerging suite of studies detailing the involvement of small singlet carbenes in driving reactions toward products of prebiotic relevance via facile addition steps.^33,57^ Given how challenging it is to detect N-heterocycles with a weak dipole moment (i.e., weak rotational transitions) directly,^16,58^ we posit that targeted searches for the precursors to N-heterocycles could reveal circumstantial evidence as to N-heterocycles’ gas-phase presence. The calculated dipole moment for **AM** using M06-2X/aug-cc-pVTZ is 3.47 D, which is stronger than pyrrole (1.86 D) and comparable to that of the recently detected aminocarbyne H_2_NC (3.83 D).^59^ Recalling the strong stabilization effect by the nitrogen’s lone pair in the singlet ground state, Eckhardt and Schreiner^14^ also calculated a long half-life for quantum mechanical tunneling from **AM** to **MA** of over 9 billion years. The results of this study position **AM** as an appropriate candidate for an astronomical investigation.

We generated the smallest aminocarbene, aminomethylene, and the resonance-stabilized propargyl radical inside a heated microreactor via pyrolysis of the precursors cyclopropylamine and propargyl iodide, respectively. A bimolecular reaction product is identified by ms-TPES as the aromatic N-heterocycle pyrrole. Semiautomated electronic structure calculations explore the C_4_H_6_N PES and show that the mechanism for pyrrole formation proceeds over submerged barriers relative to the energy of the separated reactants and terminates with the elimination of a hydrogen atom. The combination of these two mechanistic features makes this a feasible low-temperature, low-pressure pyrrole formation reaction; such characteristics hint at carbene–radical reactions as viable drivers of molecular complexity in the gas phase. The fact that the semiautomated computational investigation independently verifies the experimentally assigned reaction product is compelling evidence that the title reaction leads to pyrrole formation. Moreover, the agreement between the potential energy surface exploration and the experiment reinforces the power of a complementary approach for the treatment of analogous reaction systems. Such synergies between experiment and theory are to be exploited in the pursuit of understanding bimolecular carbene chemistry in a broad variety of environments.

## Supplementary Material



## Notes

Details of experimental and computational methods, the characterization of the reactant propargyl radical by photoion mass-selected threshold photoelectron spectroscopy (ms-TPES), a discussion of the dissociative ionization and unimolecular decomposition (pyrolysis) chemistry of the carbene precursor cyclopropylamine, the computational results and discussion regarding a reaction between methanimine and propargyl radical, additional information on the pyrrole formation mechanism, including the association step and PES plots from the KinBot single-step reaction search calculations, a statistical analysis comparing the rates of TS1–2 and TS1–7, and a table of C_4_H_5_N isomers and their ionization energies; other tables and figures (PDF)
