# Supplementary material for: Pyrrole without Life: Reaction of Aminomethylene with the Propargyl Radical
Source: J Phys Chem Lett. 2026 Feb 4;17(7):2078–84. doi: 10.1021/acs.jpclett.5c03948 (PMC12927027; doi:10.1021/acs.jpclett.5c03948)
Supplement: Supplementary file 1 [file jz5c03948_si_001.pdf]

Supporting Information to Pyrrole Without Life: Reaction of Aminomethylene with the Propargyl Radical

Rory McClish,*,†,‡ Domenik Schleier,¶ Jerry Kamer,§ Tina Kasper,‖ Andras Bodi,⊥ Patrick Hemberger,⊥ and Jordy Bouwman*,‡,†,#

†*Department of Chemistry, University of Colorado, Boulder, CO 80309, USA*

‡*Laboratory for Atmospheric and Space Physics, University of Colorado, Boulder, CO 80303, USA*

¶*Institut für Physik und Astronomie, Technische Universität Berlin, Hardenbergstrasse 36, Berlin 10623, Germany.*

§*Laboratory for Astrophysics, Leiden Observatory, Leiden University, NL 2300 RA Leiden, The Netherlands*

‖*Lehrstuhl Technische Thermodynamik, Fakultät für Maschinenbau, Universität Paderborn, Warburger Str. 100, 33098 Paderborn, Germany*

⊥*Laboratory for Synchrotron Radiation and Femtochemistry, Paul Scherrer Institut, 5232 Villigen, Switzerland*

#*Institute for Modeling Plasma, Atmospheres and Cosmic Dust (IMPACT), NASA/SSERVI, Boulder, CO 80309, USA*

E-mail: rory.mcclish@colorado.edu; Jordy.Bouwman@colorado.edu

Contents

1 Methods **S3**

1.1 Experimental S3

1.2 Computational S5

2 Additional Experimental results **S7**

2.1 Characterization of Propargyl Radical in the Pyrolysis of Propargyl Iodide . S7

2.2 Dissociative Photoionization of Cyclopropylamine S8

2.3 Cyclopropylamine Pyrolysis S9

3 Computational results **S15**

3.1 Computational Study of the Reaction of Methanimine and Propargyl S15

3.2 Association Step in the AM-headPR Reaction Mechanism S26

3.3 KinBot study of the AM-headPR Reaction Mechanism S27

3.4 KinBot study of the AM-tailPR Reaction Mechanism S34

3.5 Consideration of other C_4H_5N isomers of m/z 67 . . . . S38

References **S40**

1 Methods

1.1 Experimental

Experiments were performed using the double imaging photoelectron photoion coincidence (i^2PEPICO) spectrometer endstation at the vacuum ultraviolet (VUV) beamline of the Swiss Light Source (SLS) at Paul Scherrer Institute, Villigen. The beamline characteristics and the i^2PEPICO system have been described in detail[1–3] and only a brief description of relevant parameters is given here.

Cyclopropylamine was purchased from Sigma-Aldrich ($\geq$ 99%) and used without further purification. Propargyl iodide was synthesized using a Finkelstein reaction.[4] Glass bubblers filled with their respective samples were kept at room temperature and connected to gas feed lines such that pure argon flowed through each bubbler before passing through MKS mass flow controllers (MFC) to generate a dilute flow of gas containing the reagents entrained in argon. Experiments were conducted on the individual precursor samples seeded in argon as well as on a co-flow of both. An additional line of pure argon was used to control the overall concentration and flow rate of the gas mixture fed into the pyrolysis reactor.

The gas mixture flowed through a stainless steel tube and expanded through a pinhole into the SiC pyrolysis microreactor mounted inside the source vacuum chamber. The nozzle diameters used were 100 µm and 200 µm. The pressure before the expansion was measured using a capacitance gauge in the range of 100–200 mbar. Typical flow rates for the **CPA** pyrolysis experiments were 1 sccm **CPA**/Ar merged with an additional 10 sccm Ar, resulting in a total flow of 11 sccm with a concentration of about 0.5% **CPA** in Ar. In the **CPA** + propargyl iodide co-flow experiments all three MFCs (**CPA**/Ar, propargyl iodide/Ar, and the pure Ar line) were set to 10 sccm, for a total merged flow rate of 30 sccm. The gas expanded into the microreactor and subsequently into the source chamber where the pressure was maintained at ~ 1–5×10^{-4} mbar by two 1600 Ls^{-1} Pfeiffer turbomolecular pumps.

The Chen-type SiC microreactor was 3 cm long with an inner diameter of 1 mm. The

reactor properties and flows have been studied in detail.[5–7] The microreactor was resistively heated over a length of approximately 1.5 cm by a direct current. Approximate temperatures with a relative error of about $\pm 100\,\mathrm{K}$ were determined by calibrating the dissipated power against previous thermocouple measurements. The residence time of gas flowing through the microreactor is typically a few tens of microseconds. At the characteristic pressure (ca. 10–20 mbar) and temperature (1000 K), there are thousands of collisions between species and, thus, multiple collisions between reactive species, too.[5]

Unimolecular thermal decomposition of the precursors inside the microreactor generates radicals that initiate bimolecular chemistry. The gas mixture containing radicals, reaction products and leftover precursor species expands from the SiC reactor and is skimmed by a 1 mm diameter skimmer to form a molecular beam in the adjacent detection chamber, which is maintained at a pressure of $5–10 \times 10^{-7}$ mbar. Based on the investigation by Hemberger et al.[7] of this continuous flow experimental setup, we expect minimal rovibrational cooling within the expansion. In the detection chamber, the molecular beam is intercepted by tunable VUV synchrotron radiation produced using a bending magnet and monochromatized using a 150 grooves/mm gold-coated blazed grating. Higher harmonic monochromator radiation was filtered out using a noble gas filter for energies exceeding 10.5 eV and a MgF_2 window for energies below 10.5 eV. The light intensity of monochromatized radiation in the interaction region of the molecular beam is on the order of 10^{12} photons/s and the resulting photon energy resolution was about 1 : 1500.

Species in the molecular beam ionize upon absorbing a photon with an energy greater than or equal to its ionization energy, resulting in a photoelectron-photoion pair. The electron and ion are accelerated in opposite directions by an electrostatic field of 218 V/cm and are subsequently imaged onto RoentDek delay-line detectors. The arrival time of an imaged photoelectron serves as the start time for measuring the flight time of the corresponding photoion. At a given photon energy, time-of-flight mass spectra (PI-TOF-MS) are constructed by finding delayed coincidences of the photoelectrons, regardless of their position on the

detector (i.e., their energy) and photoions.

Selecting only the electrons with close to zero kinetic energy (i.e. threshold electrons imaged near the center of the detector) and plotting the ion counts coincident with these electrons for a given m/z as a function of photon energy results in a photoion mass-selected threshold photoelectron spectrum (ms-TPES). Both the false coincidence background and contributions from hot electrons (kinetic energy electrons detected in the center because they have zero initial lateral momentum) were accounted for by the procedure from Sztaray et al.[8]

Mass spectra were recorded at various photon energies and temperatures to obtain a holistic view of the chemistry inside the microreactor for **CPA** in argon, propargyl iodide in argon, and for the co-flow of the two radical precursors. Threshold photoelectron spectra were recorded by scanning the photon energy in steps of 10 meV using an integration time of 180 s per point. The ms-TPES provides insight into the electronic structure of the species of the selected m/z value. Reference data and/or simulations of the ms-TPES are used for isomer-specific identification of the products emanating from the pyrolysis microreactor.

1.2 Computational

The C_4H_6N potential energy surface (PES) was explored to gain insight into the chemical reactions at play in the microreactor. Electronic structure calculations were performed using the Gaussian16 computational chemistry software[9] on CU Boulder's Research Computing facility. In order to gain a complete view of the PES, the quantum chemical calculations were orchestrated using KinBot, an open-source python program that automatically explores the PES based on an extensive suite of predefined reaction templates starting from a user-defined initial input structure. The KinBot code has been described in detail in the literature.[10,11] The initial reaction searches are carried out at a L1 level of theory set at B3LYP/6-31G[12,13] before optimizations of molecular equilibrium geometries and calculations of harmonic vibrational frequencies were recalculated at the L2 level set to M06-2X/aug-cc-pVTZ.[14,15] For

each KinBot reaction search, the user-defined threshold for reaction barrier heights was typically restricted to pathways with TS energies beneath the sum of the reactants' energy, but high enough to capture multiple reaction steps necessary to ascertain the TS with the lowest relative energy, typically on the order of 200 kJ/mol.

Due to the size of the C_4H_6N doublet potential energy surface, we typically ran a series of KinBot jobs in which the starting structure was a C_4H_6N radical connected to the reaction path. For example, in the study of the **AM + PR** mechanism study, we started first with KinBot jobs on the two initial C_4H_6N intermediates resulting from the possible configurations of **AM + PR** association and calculated the complete set of connecting reaction steps for both with KinBot. In each case, by examining all of the reaction steps connected to a given input, we identified the TS with the lowest relative barrier height and followed it to the attached local minimum that was then used as an input for the next KinBot job. Generally, we repeated this process until the lowest energy step resulted in lighter products. A similar workflow was followed for the **MA + PR** study (below). Additionally, select other wells were also explored with KinBot where necessary.

This approach is chosen to balance the computational cost of exploring a wide region of the C_4H_6N potential surface with facilitating ample understanding of the relative energetics for the single-step reactions connected to a given local minimum. Hence it enabled pursuing a broad overview of the **AM + PR** reaction and thus finding the minimum energy pathway of the **AM + PR** reaction. We also employed KinBot to investigate the reaction pathways of potential side reactions like that of **MA + PR** (see the dedicated section below in the SI). The results of each KinBot job may be visualized with the open-source python script *PESViewer*.[16]

For straightfoward homolytic scissions identified by KinBot, where a C_4H_6N radical intermediate breaks to bimolecular products (often $C_4H_5N + H^{\cdot}$), we simply include the combined energy of the products and do not further treat the possibility of reverse barriers (paths shown as dotted gray lines on the PES in the SI figures). Adiabatic ionization energies are calcu-

lated at CBS-QB3 where necessary for molecules without measured ionization potentials reported in the literature.

The lowest energy conformer found for the selected stationary points were further refined with single-point electronic energies using the coupled-cluster method with single, double, and perturbative triple excitations, CCSD(T),[17] with the Dunning aug-cc-pVTZ basis set. The energies are summed to include the M06-2X zero-point corrections throughout. Where necessary, bimolecular association steps were manually examined by conducting relaxed bond length scans. Optimized transition states were verified by examining the imaginary normal mode and intrinsic reaction coordinate (IRC) calculations. For the kinetics analysis we used the DFT optimized geometries and normal mode frequencies, as well as the barrier heights at the CCSD(T) level, to calculate the forward rate constants using the transition state theory as described by the Rice-Ramsberger-Kassel-Marcus (RRKM) method within the MiniPepico program.[18]

Ms-TPES were assigned by comparison with reference data from the literature wherever possible. In addition, unless otherwise noted, Franck-Condon factors were calculated with Gaussian 16[9] in the double harmonic approximation at the M06-2X/aug-cc-pVTZ level of theory. The resulting stick spectrum was convolved with a Gaussian profile with a full width at half maximum (FWHM) of 40 meV to account for the rotational envelope and facilitate comparison with the experimental ms-TPES.

2 Additional Experimental results

2.1 Characterization of Propargyl Radical in the Pyrolysis of Propargyl Iodide

Propargyl iodide was diluted in argon and flown through the pyrolysis microreactor kept at $\approx$ 930 K. A strong ion signal was observed in the VUV photoionization TOF mass spectrum at m/z 39 and the ms-TPES of this signal is shown in Fig. S1. Comparison to the reference

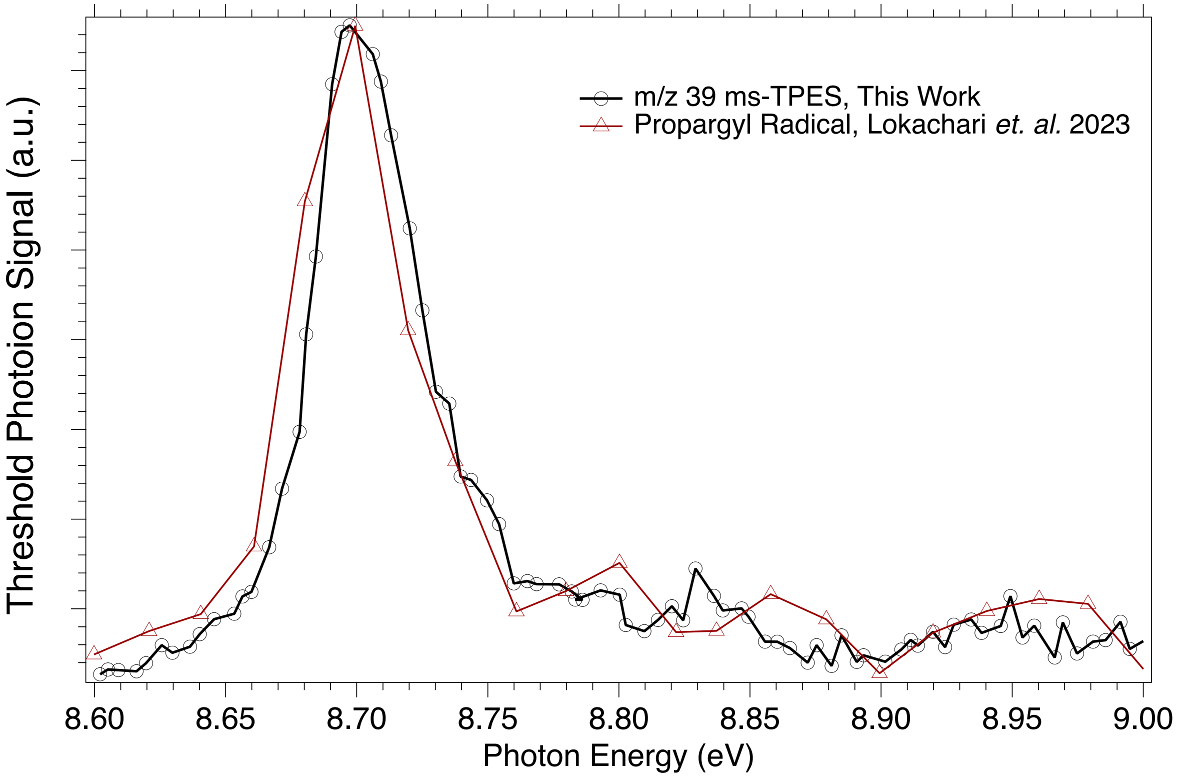

Figure S1: ms-TPES of the m/z 39 signal formed upon pyrolysis of propargyl iodide (black, circles) shown together with the reference spectrum of propargyl (red, triangles).

spectrum of propargyl enables a clear assignment.[19]

2.2 Dissociative Photoionization of Cyclopropylamine

The dissociative ionization of **CPA** has been explored previously in the works of Bouchoux and coworkers[20,21] and Papp *et al.*[22] These electron impact and photoionization studies describe a suite of dissociation pathways open at high energies and report that, up to 10.5 eV, the dominant ions are $C_3H_7N^+$ and $C_3H_6N^+$ attributed to the parent ion of **CPA** and the H-loss fragment ion, respectively.[20]

To make sure fragment ions from dissociative photoionization (DPI) of the parent **CPA** do not interfere with the interpretation of the pyrolysis data, we recorded photoionization mass spectra from 8.0 to 10.5 eV in steps of 0.5 eV without heating the microreactor. The mass spectra are displayed in Fig. S2 and confirm the results of previous studies in that only

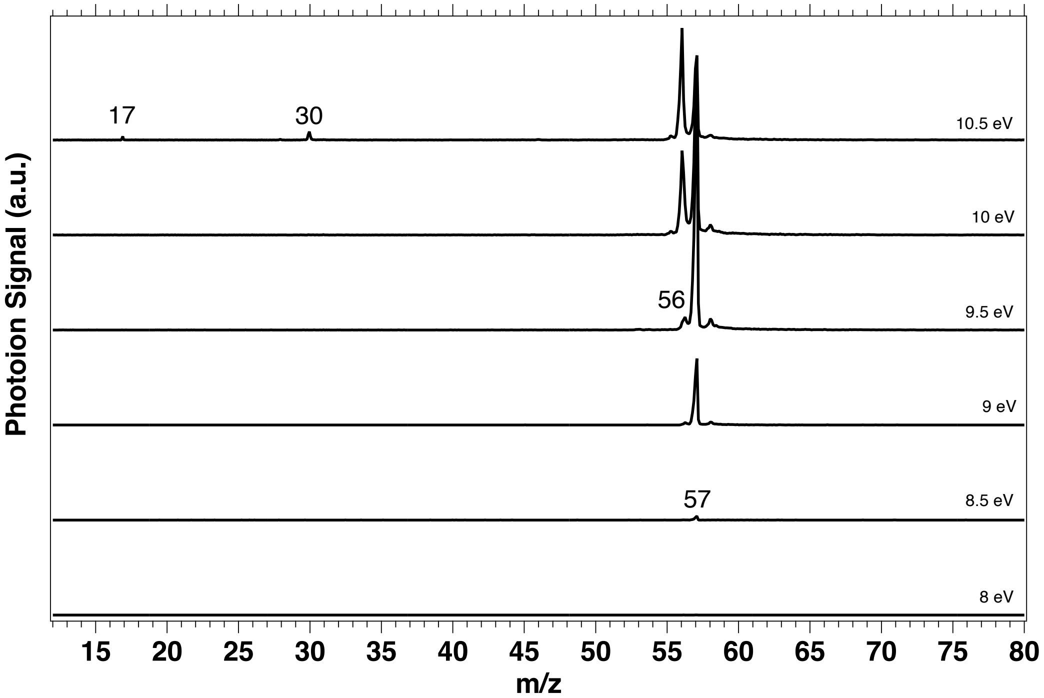

Figure S2: Stack plot of the (dissociative) photoionization time-of-flight mass spectra of cyclopropylamine seeded in argon collected at selected photon energies

ions with m/z 58, 57, and 56 are detected below 10 eV. The two peaks at m/z 58 and 57 are due to ionization of the ^{13}C isotopologue of $C_3H_7N^+$ and parent **CPA**, respectively, while the first DPI fragment corresponding to H-loss appears at m/z 56. In the 10.5 eV spectrum, we characterize the ion signal at m/z 17 as neutral ammonia, which is present in trace amounts as a contaminant in the ionization chamber. The peak at m/z 30 is a DPI product attributed to $H_2C{=}NH_2^+$ as assigned by Papp and coworkers with a measured appearance energy of 9.98±0.2 eV.[22] Fig. S2 shows that DPI is a minor process below a photon energy of 10 eV.

2.3 Cyclopropylamine Pyrolysis

We studied the thermal decomposition of **CPA** to better optimize the production of aminomethylene (**AM**). Photoionization time-of-flight mass spectra were collected at two photon energies (9 and 10 eV) and at various pyrolysis powers, the results of which are shown in Figs S3 and S4. In the 9 eV mass spectrum (Fig S3), temperature-dependent ion signals are seen at

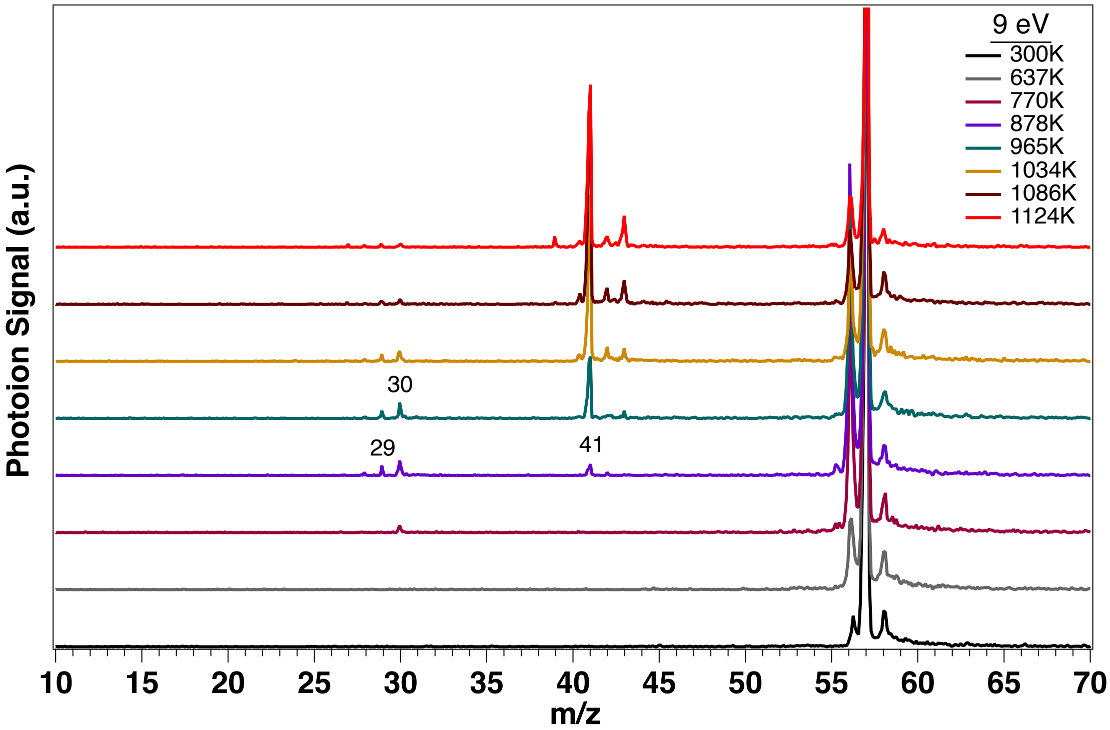

Figure S3: Stack plot of PI-TOF-MS recorded at 9 eV of **CPA** seeded in argon as a function of the wall temperature of the SiC microreactor

m/z 28, 29 30, 39, 41, 42, 43, 56, 57, and 58. The mass spectra recorded at 10 eV (Fig S4) reveal additional peaks at m/z 15, 17, 27, 31, 40, 54, and 55.

Previous studies of the pyrolysis of **CPA** have primarily focused on the isomerization of **CPA** to open-chain C_3H_7N products as well as their subsequent reactions with a second **CPA** molecule.[23–27] However, both the works of Hamada and Tsuboi[25] and Eckhardt and Schreiner[28] report observation of lighter products formed from unimolecular decomposition through the use of infrared spectroscopy experiments. The combined literature details the identification of CH_3 (m/z 15), CH_4 (m/z 16), NH_3 (m/z 17), HCN (m/z 27), C_2H_4 (m/z 28), $HCNH_2/H_2CNH$ (m/z 29), and the C_3H_7N isomers 1-aminopropene (E and Z) and allylamine (m/z 57). Of these, we can only verify those with ionization energies below the photon energy, i.e. contributions of methane (IE= 12.61 eV),[29] hydrogen cyanide (IE= 13.60 eV),[30] and ethylene (IE= 10.51 eV)[31] are not observed in our experiments. This leaves the signals at m/z 27, 30, 31, 39, 40, 41, 42, 43, 54, 55, and 56 seen in our spectra as

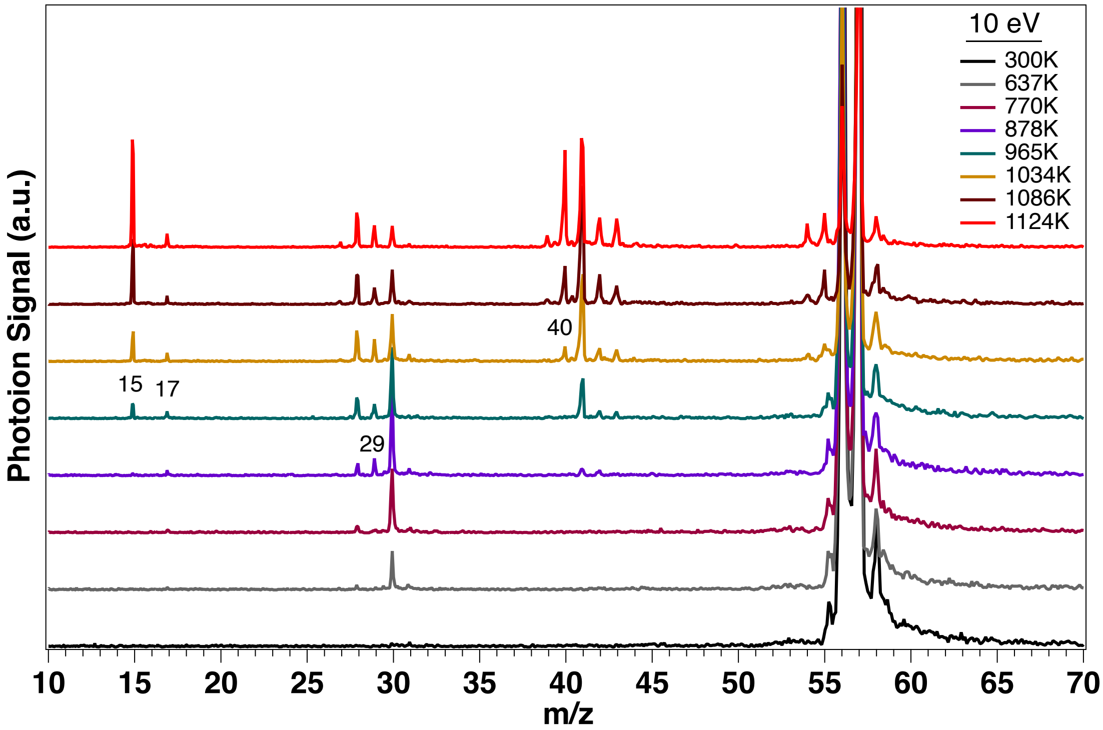

Figure S4: Stack plot of PI-TOF-MS recorded at 10 eV of **CPA** seeded in argon as a function of the wall temperature of the SiC microreactor

previously unreported species formed from the thermal decomposition of **CPA**.

Based on the subsequent co-flow experiments, the **CPA** pyrolysis products formed below 1000 K are all of interest. Under such conditions, the peaks at m/z 29, 30, 41, 43, and 56 all originate from species with ionization energies below 9 eV (below the yellow trace, Fig. S3). Based on a calculated IE of 6.22 eV for the H_2CNH_2 radical, the m/z 30 ion peak can be tentatively attributed to H_2CNH_2 radicals that can be formed by, e.g., H-addition to **AM**. The ion peak at m/z 43 is thought to originate from vinylamine ($C_2H_3NH_2$) considering it has an IE of 8.20 eV.[32] We speculate that a methyl radical adds to **AM** and subsequent β-scission is responsible for the formation of vinylamine. In the case of m/z 41, the vibrationally resolved ms-TPES matches the reference spectrum for allyl radical as shown in Fig. S5.[7] The allyl radical is likely formed through the loss of $NH_2^{\cdot}$ from **CPA** or a C_3H_7N isomer. The amino radical itself is not observed at this photon energy due to its ionization energy of 11.14 eV.[33] At least some of the counts at m/z 56 are attributed to DPI, and the DPI

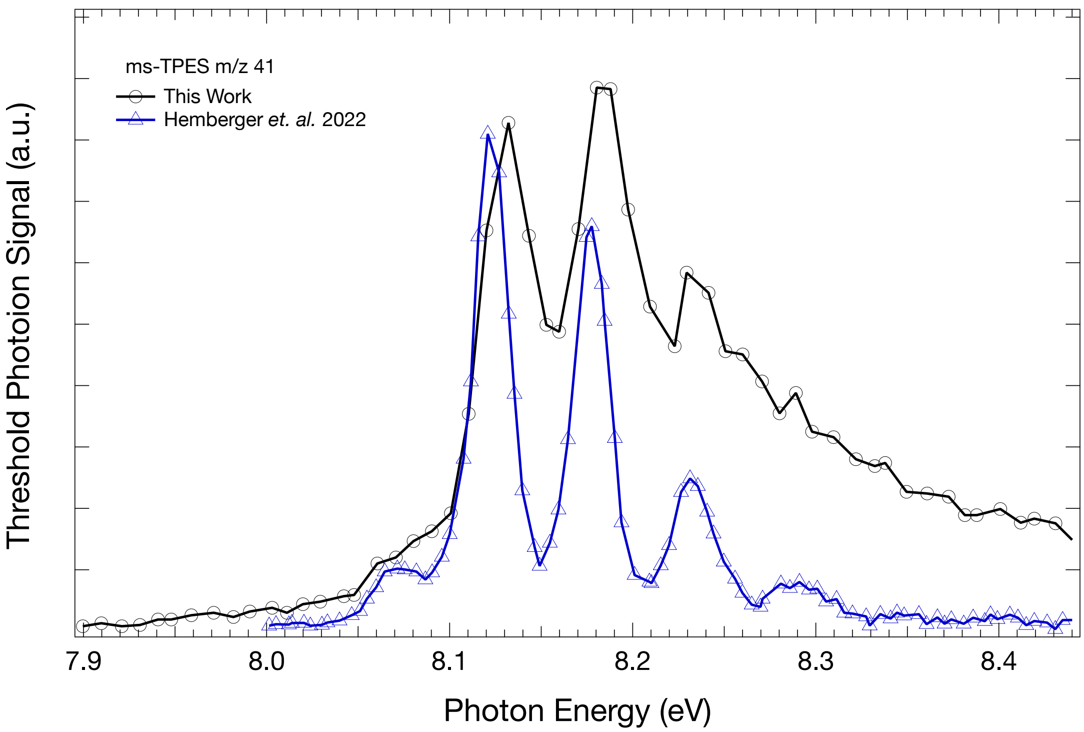

Figure S5: The ms-TPES of m/z 41 collected in this study (black) compared with the allyl radical ms-TPES (blue) recorded by Hemberger et al.[7]

fragment appearance energy may red shift with pyrolysis, increasing the signal intensity in the 9 eV pyrolysis experiment. Still, here we do not rule out an H-loss thermal decomposition product from a C_3H_7N species.

The peaks at m/z 28, 40, and 55 and are only observed in the 10 eV mass spectra (Fig. S4). While ethylene is a known decomposition product of m/z 28, it has an IE of 10.5 eV,[31] which is too high for it to contribute to the 10 eV spectrum. Since the peak is largely absent in the 9 eV spectrum, we suspect that it corresponds to a CNH_2 isomer with an IE between 9 and 10 eV, potentially the methylene amidogen radical (H_2CN, IE = 9.4 eV).[34] The peak at m/z 40 is unassigned, as multiple C_3H_4/H_2C_2N isomers with IEs in the range 9–10 eV could contribute. Finally, no m/z 55 was observed in the DPI study, and so is suspected to be an H_2-loss pyrolysis product from the neutral parent **CPA**.

As pointed out in the main text, the 9 eV mass spectrum in Fig. S3 does not contain ion signal from **MA** due to its IE of 9.99 eV.[36,37] The intensity of the m/z 29 ion peak,

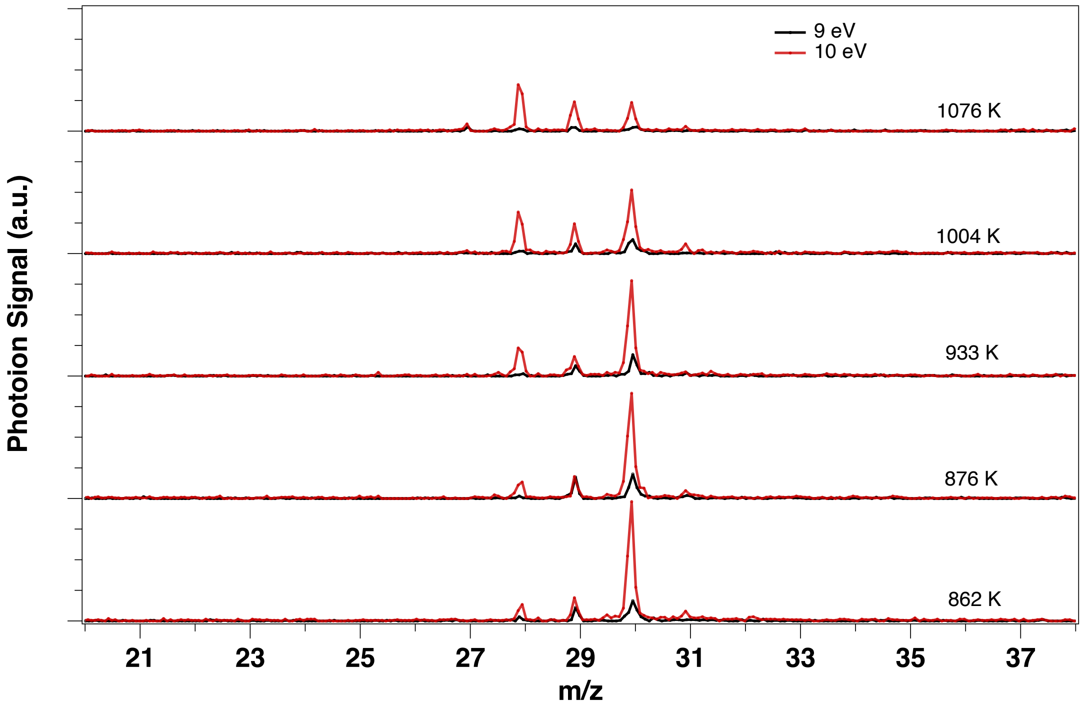

Figure S6: Stack plots of PI-TOF-MS recorded at both 9 eV (black trace) and 10 eV (red trace) of **CPA** seeded in argon as a function of the wall temperature of the SiC microreactor. The mass spectra is the same data plotted in Figs. S3, S4 above.

thus attributed exclusively to **AM**, reaches its maximum near 880 K. It then decreases and vanishes almost entirely at pyrolysis temperatures near 1150 K. This observation supports the notion that **AM** is synthesized with an appreciable lifetime and is stable enough towards thermally induced hydrogen migration to methanimine up to ~ 1000 K. In the 10 eV mass spectrum (Fig S4), however, the m/z 29 peak does not decrease at higher temperatures. This can be explained by **AM** isomerizing to the more stable **MA**, as the higher temperature lifts its internal energy distribution above the isomerization barrier sufficiently so that thermal equilibrium can be approached within the residence time in the microreactor. We thus determine that the **AM** abundance is highest when the wall temperature is near 900 K.

The interplay of **AM** and **MA** is important from the perspective of assigning the reactant in the co-flow experiment described in the main text. The mass spectra in Figs S3 and S4 are overlapped in Fig. S6 to better compare the specific behavior of the m/z 29 peak as a function of temperature. First, the aforementioned observation of m/z 29 disappearing

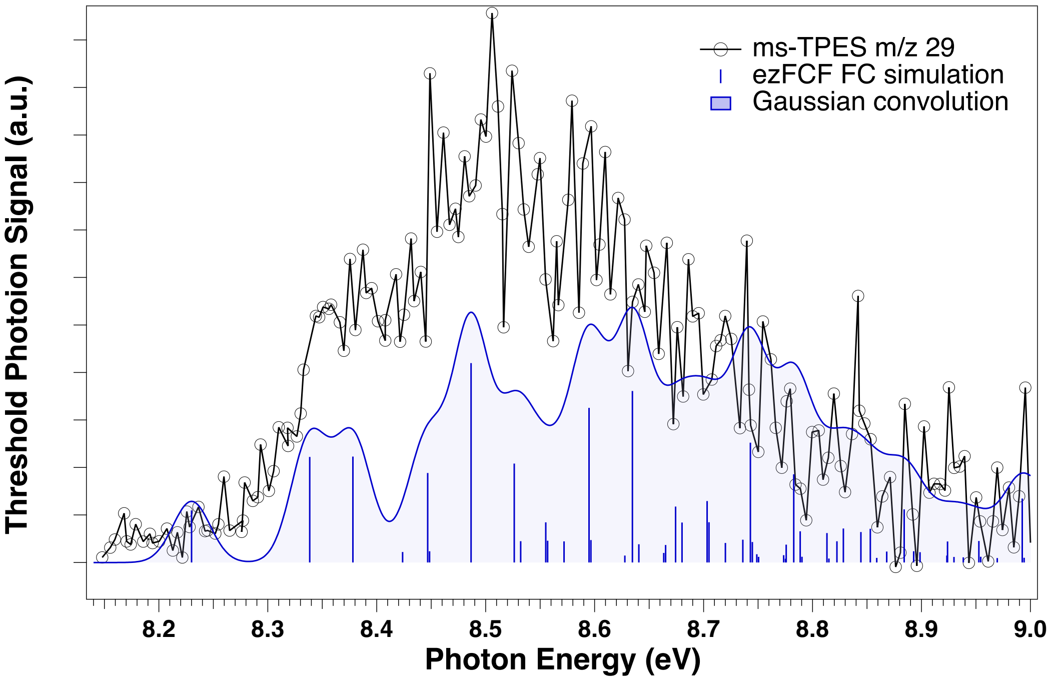

Figure S7: The mass-selected threshold photoelectron spectrum of m/z 29 between 8.15–9.0 eV collected in steps of 5 meV. A 1 sccm flow of **CPA** seeded in Ar is merged with a 19 sccm flow of pure Ar and the SiC microreactor heated to approximately 430 K. A FC simulation stick spectrum using the code ezFCF[35] is included in blue with the shaded Gaussian convolution.

at high temperatures in the 9 eV spectra versus holding relatively stable as a function of temperature in the 10 eV spectra is apparent. Second, the near match in overlaid peaks circa 876 K is evidence that at these lower pyrolysis temperatures not only is **AM** production optimized, but also that **MA** is not appreciably abundant. The selection of these "**AM**-favored" pyrolysis conditions for the subsequent co-flow experiments discussed in the main text is helpful in minimizing any contribution from side reaction with **MA**.

Aminomethylene was first detected in collisional activated dissociation and neutralization-reionization mass spectrometry investigations.[38,39] Its infrared spectrum was recorded using matrix-isolation IR spectroscopy to probe the pyrolytic mixture of **CPA**.[28] In this work, we recorded the m/z 29 ms-TPES of **AM** in the 8.1–8.9 eV photon energy range (Fig. S7). The onset of the signal is in agreement with the calculated CBS-QB3 AIE of 8.23 eV corresponding to the transition from the singlet neutral to doublet cation ground state. The observed

band structure is qualitatively reproduced by the vibronic progressions in the Franck-Condon simulation, but individual transitions are not resolved. The simulated band is broader and decays at higher photon energies, which is characteristic of an anharmonic cation potential, leading to a crowding of the vibrational levels as the energy is increased.

Triplet **AM** is calculated to lie 1.5 eV higher in energy than the singlet. A π-donor effect from the lone pair of the nitrogen to the empty p orbital on the carbenic carbon stabilizes the singlet. Furthermore, in the concerted chelotropic reaction to form **AM**, the triplet is not expected to be a primary product in the unimolecular decomposition of the closed-shell **CPA**. The calculated ionization potential of the triplet is 6.7 eV, which falls well below the observed ion signal onset of 8.2 eV in the ms-TPES of m/z 29. We thus do not observe the triplet carbene, and the low numbers of possible isomers for CNH_3 help confirm the identification of the smallest aminocarbene.

3 Computational results

3.1 Computational Study of the Reaction of Methanimine and Propargyl

The reaction between aminomethylene's isomer, methanimine (**MA**), and **PR** may represent an alternative mechanism to form pyrrole. Because of the higher stability of **MA** with respect to **AM** (in the neutral case), this reaction could even be more relevant in a number of chemical environments. In considering the possible initial intermediates from association of **AM** and **PR**, the carbenic carbon in **AM** offers a clear reactive site to react with propargyl. However, either resonance form of **PR** (head/tail) could also add to the π-bond connecting the carbon and nitrogen atom in **MA**, thus yielding four possible association reactions, all of which yield radical C_4H_6N isomers that might lead to pyrrole and hydrogen loss.

The four initial radical complexes formed from addition of the head/tail of **PR** to the carbon or nitrogen of **MA** are notated: **cMA-headPR** ($HNCH_2CHCCH_2$), **cMA-tailPR**

($HNCH_2CH_2CCH$), **nMA-headPR** ($H_2CNHCHCCH_2$), and **nMA-tailPR** (H_2CNHCH_2CCH) (see Fig. S8). We locate the four transition states for the initial bimolecular association of $H_2CN+C_3H_3$ to lie between 48 and 70 kJ/mol above the energy of **MA+PR**. Visualizing the geometry along the association reaction coordinate demonstrates that the transition state is due to the elongation of the CN bond distance in **MA**. Specifically, in the case of the formation of both **cMA-PR** intermediates (nearly isoenergetic at **cMA-headPR** at -187.8 kJ/mol and **cMA-tailPR** at -187.9 kJ/mol), the entrance barriers from **MA+PR** are 59 and 48 kJ/mol, respectively. The analogous association barriers for propargyl to bond to the imine-end of **MA**, as is the case for **nMA-headPR** and **nMA-tailPR**, are calculated to be slightly higher at 70 and 61 kJ/mol, respectively. The summary in Fig. S8 shows the connection of the **cMA-PR** channels to the common potential wells **I1** and **I4** in the portion of the PES already explored in the **AM-PR** study. The reader should note that in the summary PES (Fig. 3 in the main and Fig. S8 here), the low number of stationary points permitted treatment with CCSD(T)/aug-cc-pVTZ//M06-2X/aug-cc-pVTZ, while in the following KinBot plots the highest level of theory is the pure DFT sum of electronic and ZPE at M06-2X/aug-cc-pVTZ. In Figs. S9, 10, 11, and 12, the forward barrier for **MA + PR** are not calculated by KinBot, and so we incorporate our 'manually' calculated TS at M06-2X/aug-cc-pVTZ into the plots and highlight this entrance channel in orange.

The **cMA-PR** intermediates are intuitively more likely to connect to the portion of the PES associated with the reaction of **AM-PR** based on the identical heavy-atom skeleton. For each of the initial **cMA-PR** wells, the single-step KinBot results are visualized in Figs. S9 and S10. Intermediate **cMA-headPR** (Fig. S9) might be expected to be directly connected to **I2** on the main **AM+PR** PES via a 4-membered cyclic intermediate, but neither Kinbot reaction templates nor our 'manual' attempts using relaxed potential energy surface scans, nor the STQN method, found a single-step isomerization pathway between intermediates **cMA-headPR** and **I2**. However, hydrogen transfer from the C1 position to the nitrogen resulting in **I1** is accessible over a barrier of 131 kJ/mol (Fig. S8, TS at −56.5 kJ/mol).

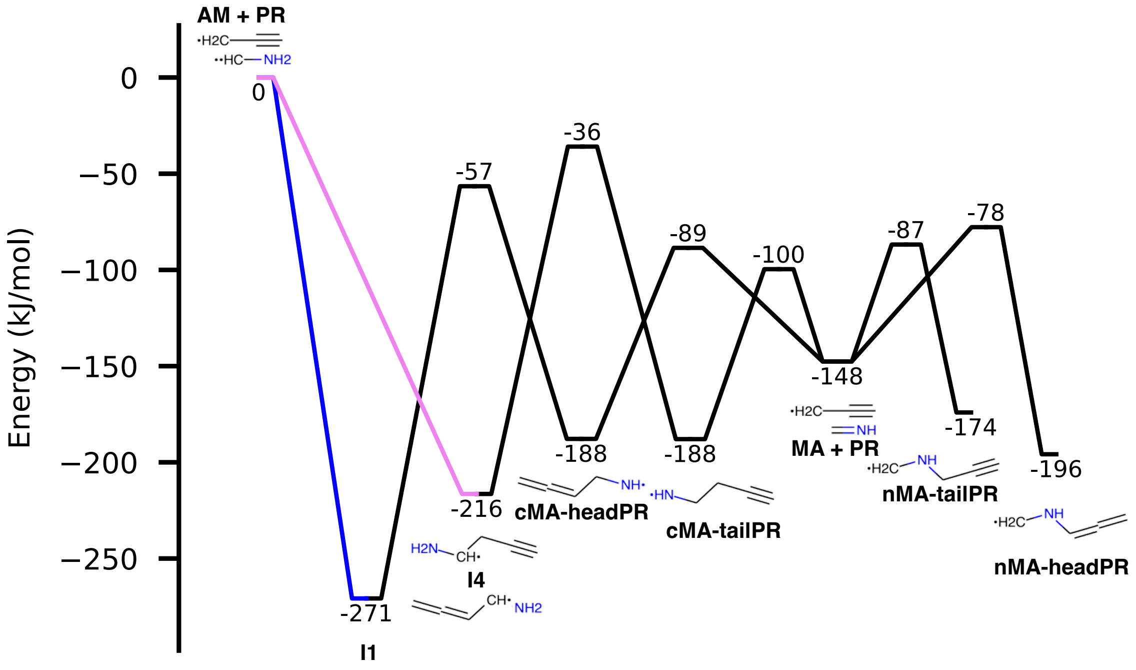

Figure S8: MA+PR initial intermediates and the connection to the AM+PR portion of the C_4H_6N PES, in which the DFT geometries and ZPEs are used with single point energies from CCSD(T)/aug-cc-pVTZ

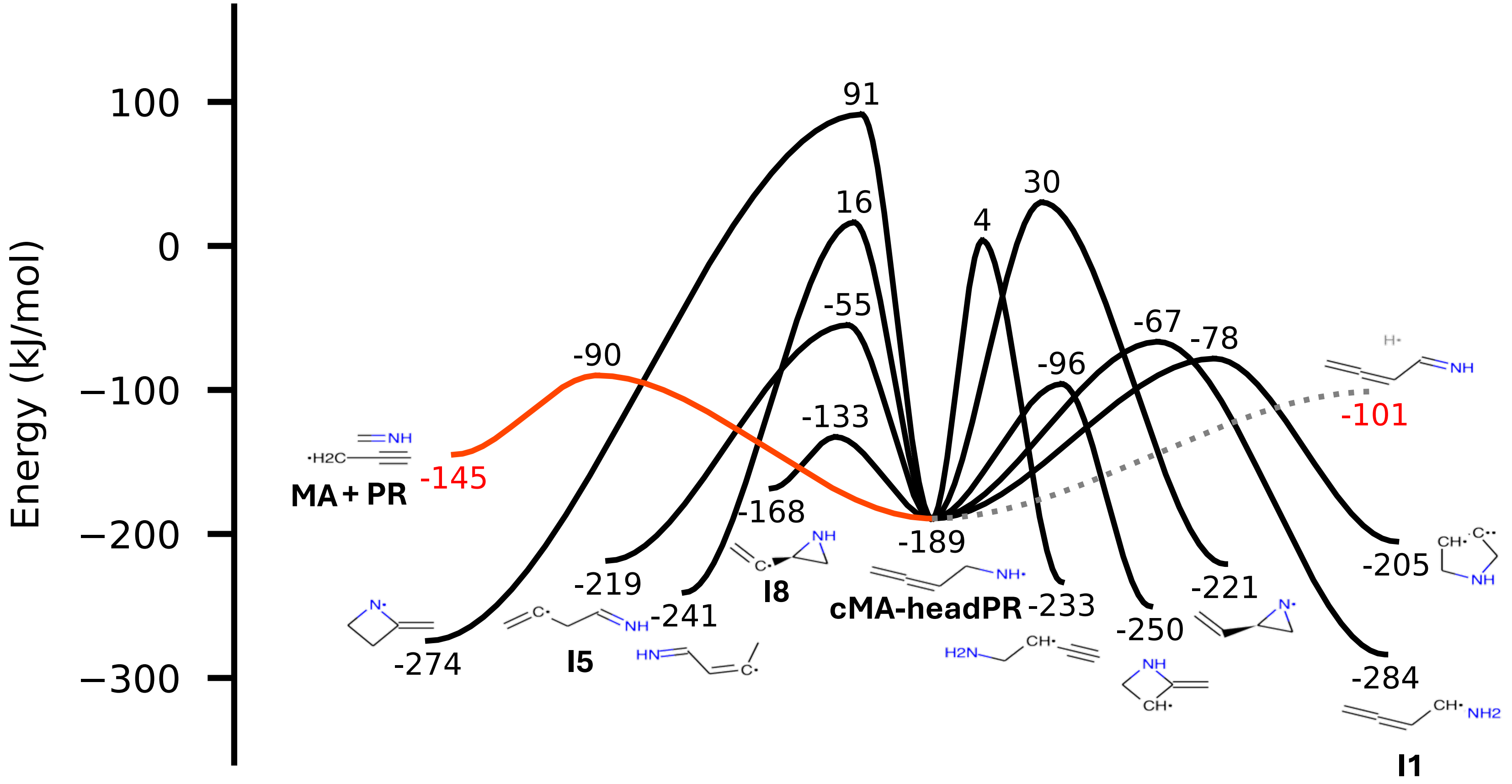

Figure S9: KinBot calculation at M06-2X/aug-cc-pVTZ starting from cMA + headPR

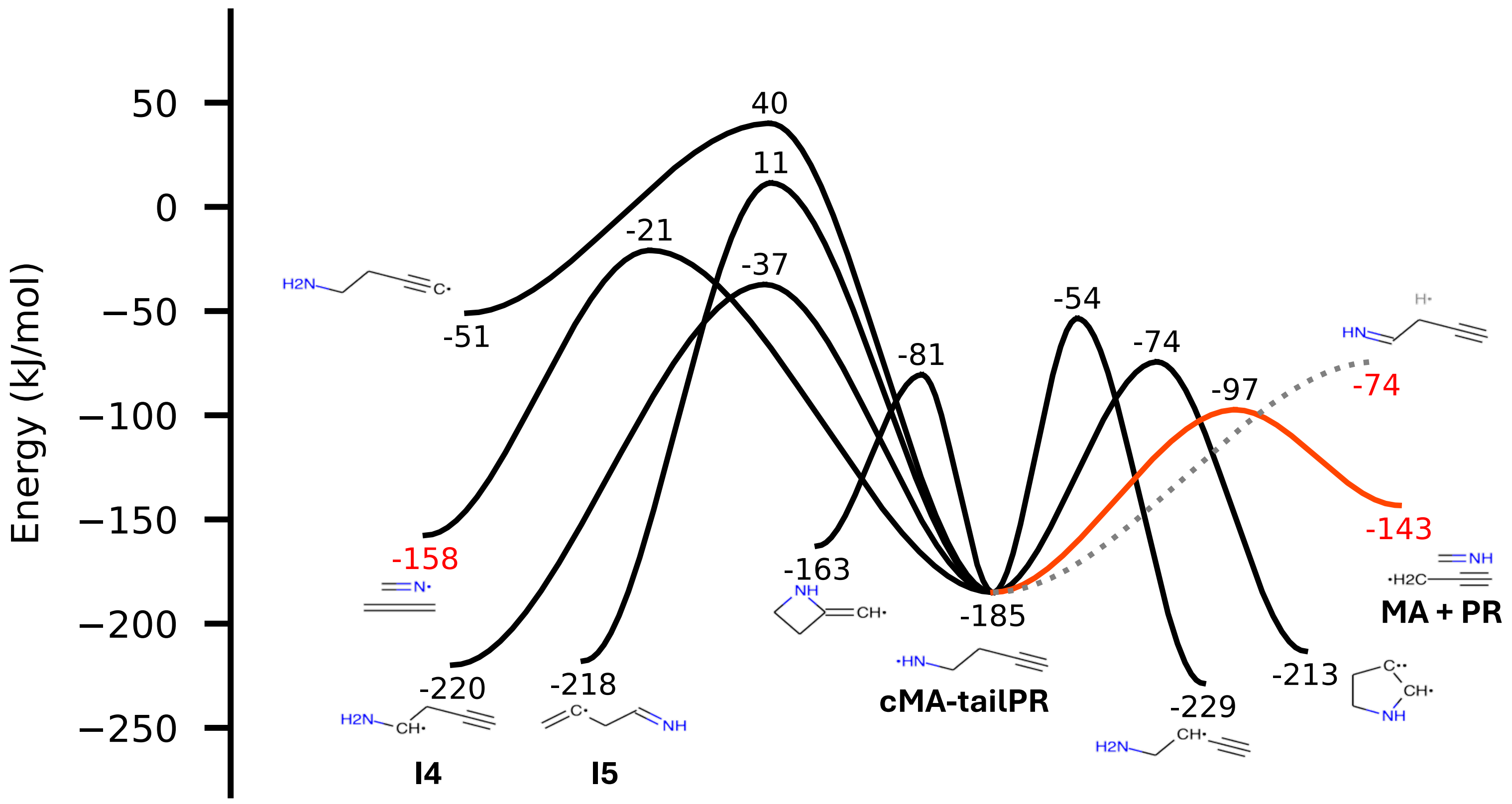

Figure S10: KinBot calculation at M06-2X/aug-cc-pVTZ starting from cMA + tailPR

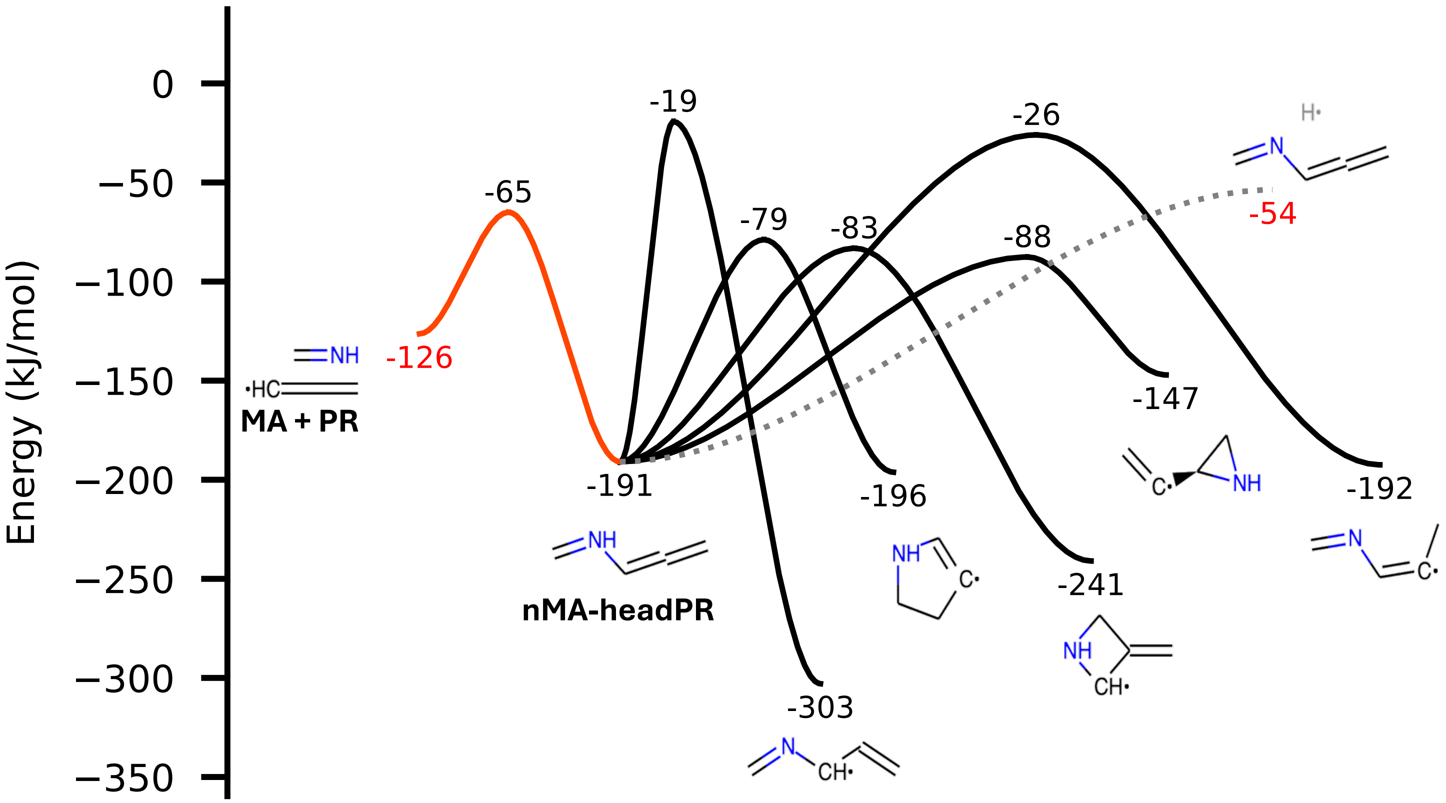

Figure S11: KinBot calculation at M06-2X/aug-cc-pVTZ starting from nMA + headPR

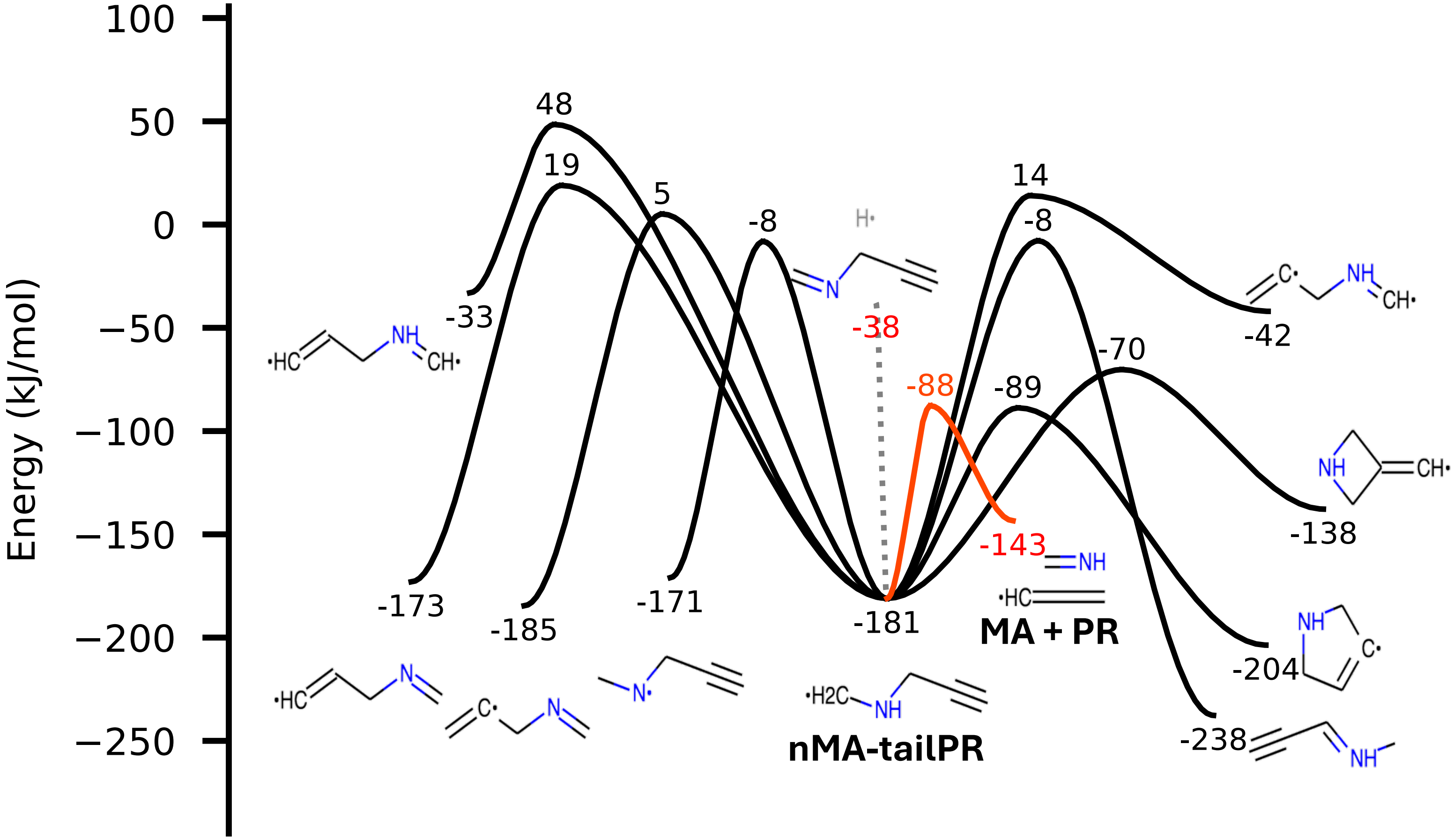

Figure S12: KinBot calculation at M06-2X/aug-cc-pVTZ starting from nMA + tailPR

The KinBot job for **cMA-headPR** (Fig. S9) also finds the hydrogen transfer to **I5**. If the dissociation of **cMA-headPR** back to reactants **MA+PR** can occur without a barrier, then even if the **MA+PR** reaction occurs, it would be expected to be kinetically driven back towards reactants. However, two cyclization reaction steps, the lowest barrier pathway leading towards the substituted 3-membered cycle shown at −168 kJ/mol in Fig. S9 and the next lowest to a substituted 4-membered ring species, both have TS energies beneath the entrance channel TS of **MA+PR**. Neither of these two steps are found to lead to favorable pyrrole formation. In the case of the $-CH_2$ side of **PR** adding to the C-terminus of **MA**, the KinBot job for **cMA-tailPR** (Fig. S10) finds the hydrogen migration step to **I4**, but the barrier in this case is higher (152 kJ/mol above **cMAtPR** in Fig. S8). The **cMA-tailPR** intermediate is also connected directly to **I5** common to the **AM-tailPR** minimum energy pathway as well, but KinBot finds no reaction steps with TS below the entrance channel barrier for **MA+PR**, making back dissociation of **cMA-tailPR** to **MA+PR** the expected fate of this intermediate. Similar to the case of **cMA-headPR**, **nMA-headPR** has reaction steps with TS energies beneath that of the corresponding addition TS of **MA+PR**, as seen in Fig. S11. These are all ring-closing steps, with the lowest barrier option towards another substituted 3-membered ring species, a 'dead end' for pyrrole formation. This is possibly the case for **nMA-tailPR** as well, Fig. S12, in which the sole competitive isomerization reaction step is the formation of a 5-membered ring that proceeds over a TS nearly isoenergetic with the TS for the addition of **MA+PR**. The expected uncertainty in DFT calculated energetics makes even the relative ordering of these two barriers uncertain, but we note that the hydrogens are not evenly distributed around the 5-membered cycle shown at −204 kJ/mol in Fig. S12. As a check, we fed KinBot this structure to find that upon ring closure, H-migrations around the ring become energetically expensive, and that re-opening back to **nMA-tailPR** is the reaction step with the lowest barrier. Seen most clearly in the summary plot Fig. S8, it is immediately clear that neither **cMA-PR** complex would be expected to connect to the **AM-headPR** or **AM-tailPR** pathways. For the three cases of **cMA-**

headPR, nMA-headPR and **nMA-tailPR**, multiple pathways are lower in energy than those that might lead to pyrrole, while in the case of **cMA-tailPR** the predicted outcome is straightforwardly the irreversible dissociation back to reactants **MA** and **PR**.

Recognizing that the physical conditions within the SiC microreactor are different than those of the ISM, we set out to obtain a first-order estimate of the difference in reactivity of **AM** and **MA**. While entrance barriers negate the astrophysical relevance of the reaction between **MA** and **PR** under the constraints imposed by cold dark molecular clouds, such barriers might be surmounted in the pyrolysis microreactor. First, we examine the effect of the **MA-PR** entrance barrier vs. the barrierless association of **AM-PR**. Within an Arrhenius picture and at an approximate gas temperature of 1000 K in our microreactor, the rate constant of **MA+PR** is:

$$\begin{aligned} k_{MA-PR} &= A \exp\left(\frac{-\Delta E}{k_B T}\right) \\ &= A \exp\left(\frac{-48000\,\frac{J}{mol} \times \frac{1\,mol}{6.022\times10^{23}}}{1.38\times10^{-23}\,\frac{J}{K} \times 1000\,K}\right) \\ &= A \times 322.4 \end{aligned} \tag{1}$$

Where A is the pre-exponential factor (the attempt frequency), ΔE is the lowest of the four TS' for the **MA-PR** reaction (48 kJ/mol), k_B is the Boltzmann constant, and T is the reaction temperature. Neglecting further effects encompassing dynamics and branching, and also assuming similar capture rates, this implies that **MA** would have to be on the order of 300 times more abundant than **AM** for the overall reaction rate of **AM-PR** and **MA-PR** to be equal. Yet, the experimental results pertaining to the pyrolysis chemistry of **CPA** as discussed above (Fig S6) suggest that **MA** is not appreciably produced when the wall temperature of the SiC microreactor is near 900 K. Furthermore, while our calculations are referenced to zero-point corrected electronic energies throughout, we also compared the reaction landscape at key stationary points in terms of Gibbs free energies as shown in Fig S13. For an understanding of the influence of the experimental reaction conditions on

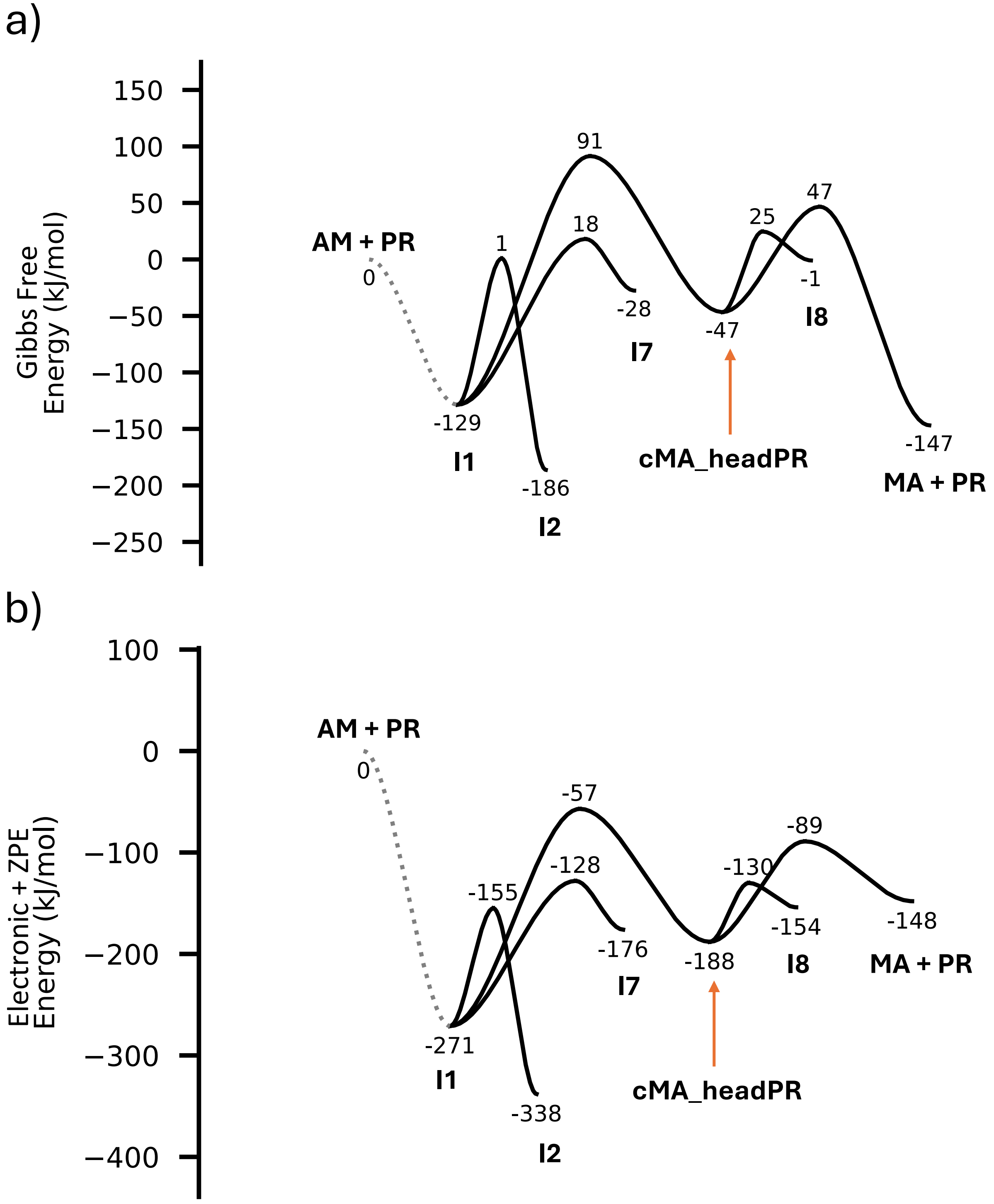

Figure S13: C_4H_6N energy landscapes comparing a) the sum of electronic energies calculated at CCSD(T)/aug-cc-pVTZ and the thermal correction to the Gibbs Free Energies at 800 K and 68 Torr calculated using M06-2X/aug-cc-pVTZ vs. b) the sum of electronic energies calculated at CCSD(T)/aug-cc-pVTZ and the zero-point corrections from M06-2X/aug-cc-pVTZ.

the relative energetics between key intermediates, the key portion of the C_4H_6N surface includes: entry points **AM+PR** and **MA+PR**, **cMA-headPR** and its lowest energy TS leading to the vinylaziridinyl radical (**I8**, Fig S9), the fifth highest energy route from **cMA-headPR** to **I1** as the connection to eventual pyrrole+H, and the two lowest energy TS' that connect **I1** to **I2** and **I7** (see dedicated **AM+PR** section below). At reasonable estimates of the reaction conditions (800 K and 68 Torr) inferred from the computational fluid dynamics study by Guan et al.[5], the relative energetics of competing reaction steps available to both intermediates **I1** and **cMA-headPR** are qualitatively consistent with the results taken from purely the sum of electronic and zero-point energies, i.e. competition between **I1** rearranging to **I2** vs. **I7** or the negligible expected branching of **cMA-headPR** to **I1**. Mainly, in the Gibbs free energy picture the association steps are predicted to be entropically penalized, notably further minimizing the chances for **MA** to promote pyrrole formation.

We conclude that while we cannot definitively rule out a contribution of the **MA+PR** reaction in our reactor, we calculate that the **MA+PR** reaction will be slower than the **AM+PR** reaction, and that more importantly it does not proceed over to pyrrole formation. The finding that the initial addition of **MA** with **PR** proceeds over a positive entrance barrier makes it less competitive with the **AM+PR** reaction. However, it is the extensive search of all of the reaction steps connected to the four possible initial intermediates of the **MA+PR** reaction that shows how rearrangements leading to the formation of pyrrole are not competitive compared to either dissociation back to the initial reactants **MA+PR** or to other $C_4H_6N^\cdot$ intermediates not known to subsequently react towards pyrrole+$H^\cdot$. Thus, it is on this theoretical basis that we exclude the **MA+PR** reaction from consideration in the mechanism resulting in the pyrrole that is sampled from our microreactor. Finally, this finding is in complement to our experimental pursuit of tuning the pyrolysis conditions of **CPA** to minimize the ion signal of **MA**.

3.2 Association Step in the AM-headPR Reaction Mechanism

We conducted relaxed PES scans at the with M06-2X/aug-cc-pVTZ level of theory (as in the KinBot PES exploration) for the association steps leading to I1 and I4 to investigate whether there is a barrier along either association path. The scan results located a first-order saddle point (**TS01**) along the association bonding coordinate between the carbenic carbon of aminomethylene and the −CH terminal of PR, as the −CCH moiety changes from linear in **PR** to bent in the adduct. This geometry serves as a starting point for optimization and frequency analysis of the TS, which were repeated using two functionals and three basis sets for comparison. We chose the ωB97X-D[40] to complement M06-2X/aug-cc-pVTZ used throughout the exploration of the C_4H_6N PES in this work; these functionals incorporate different treatments of exact exchange, range separation, and dispersion effects. The comparison was extended to include three basis sets for each functional: cc-pVTZ, aug-cc-pVTZ, and the Ahlrichs and coworkers' triple-zeta basis set def2-TZVP.[41] Finally, the M06-2X/aug-cc-pVTZ calculations were performed with and without the addition of D3 empirical dispersion by Grimme et al.[42] The relaxed scans along **AM-tailPR** leading to I4 yielded analogous results, but none of the TS calculations converged to a saddle point in which subsequent IRC calculations could confirm that the TS connected the reactants to I4. As discussed in the main text, the KinBot search beginning from I4 rules out the **AM-tailPR** pathway from contributing to pyrrole formation, so we did not further pursue this association step.

Table S1: Energy difference between TS01 and reactants (sum of electronic and zero-point energies)

	**M06-2X**				**ωB97X-D**		
**Basis Set**	cc-pVTZ	aug-cc-pVTZ	aug-cc-pVTZ + GD3	def2-TZVP	cc-pVTZ	aug-cc-pVTZ	def2-TZVP
**kJ/mol**	-2.37	-1.04	-3.32	-1.13	-1.99	-0.57	-0.82

The TS calculations of **TS01** find that the CCH angle in **PR** decreases slightly to 169°

(M06-2X/aug-cc-pVTZ) and that a single, low imaginary frequency (-234 cm^{-1}) along the bonding coordinate is dominated by the $-$CH bend of **PR**. Intrinsic reaction coordinate calculations connect **TS01** to separated reactants and the prompt **AM-headPR** association product **I1**.

The relative energetics in Table S1 corresponds to the difference between the sum of electronic and zero-point energies for the each reactant and the TS calculation. The results indicate that **TS01** is likely slightly submerged beneath (or nearly isoenergetic to) the entrance channel across all methods and basis sets explored. We attempted to refine the electronic energy of the TS with coupled cluster calculations, analogous to the process applied to stationary points found by KinBot. While the results were consistent with the DFT ones, we disregard them because of severe spin-contamination of the Hartree-Fock reference functions with $\langle S^2 \rangle \approx 1$. Thus, we conclude that at the DFT level, the association between AM and the CH end of PR is calculated to be barrierless, inclusive of a shallow, submerged TS.

3.3 KinBot study of the AM-headPR Reaction Mechanism

The mechanism following association was explored with KinBot from the initial C_4H_6N **AM-headPR** intermediate, **I1**. The results from each single-step PES calculation KinBot job are shown in Figs. S14–16, as plotted using the open-source code PESViewer.[16] In each calculation, KinBot reaction templates allow for a comprehensive 1-step exploration of reaction pathways, such that the pathways with the lowest expected rate-limiting barriers can be identified for further consideration. In each calculation, the energy threshold for the reaction search was set well above the preceeding barrier to capture multiple reaction pathways; for example, the KinBot job exploring reaction steps starting from **I2** used a barrier of 250 kJ/mol above **I2**, which is enough to capture eight reaction steps, including the hydrogen transfer between **I2** and **I1**, the ring closure to **I3**, as well as additional H-loss, cyclization, and H-transfer steps (Fig. S15). Note that the energies plotted in Figs S14–16

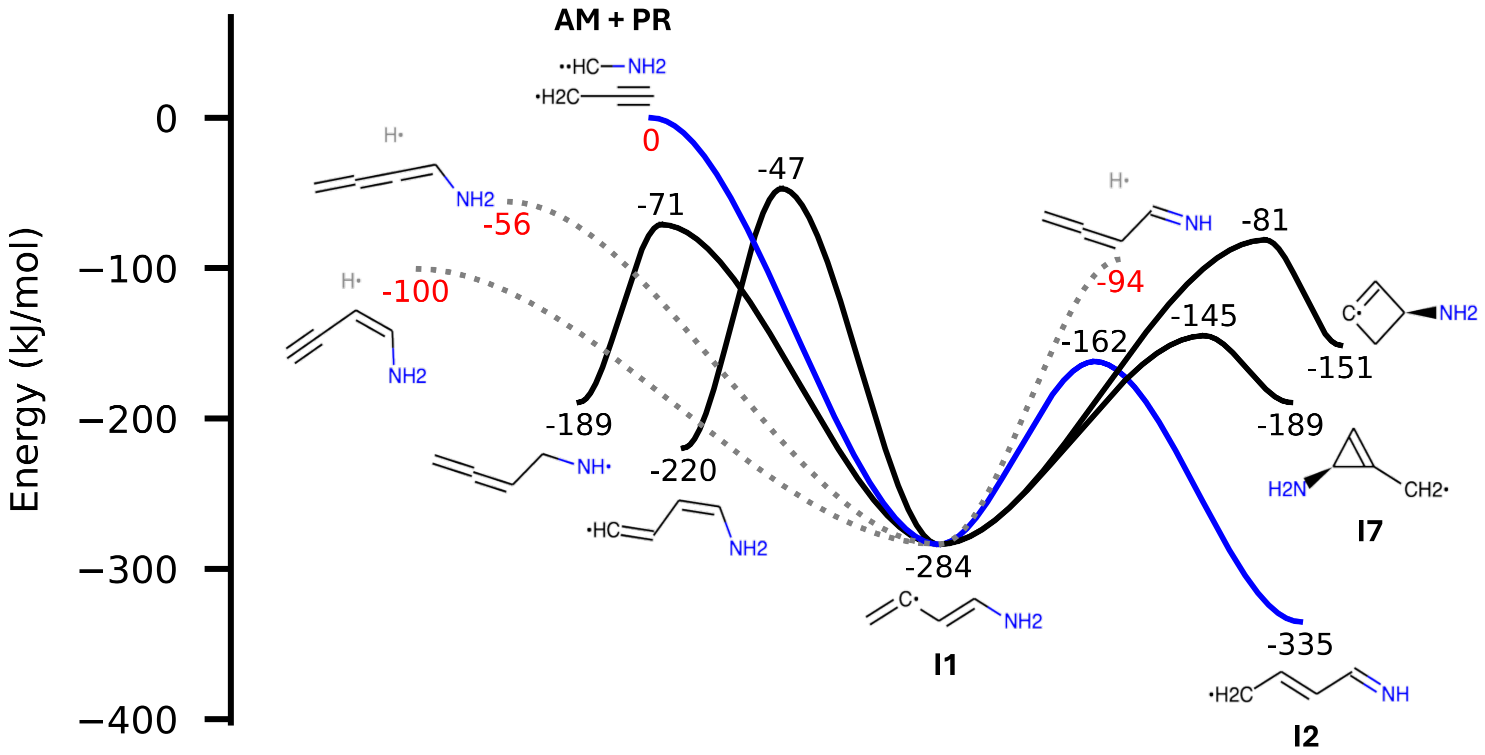

Figure S14: KinBot calculation starting from **I1**, calculated at M06-2X/aug-cc-pVTZ.

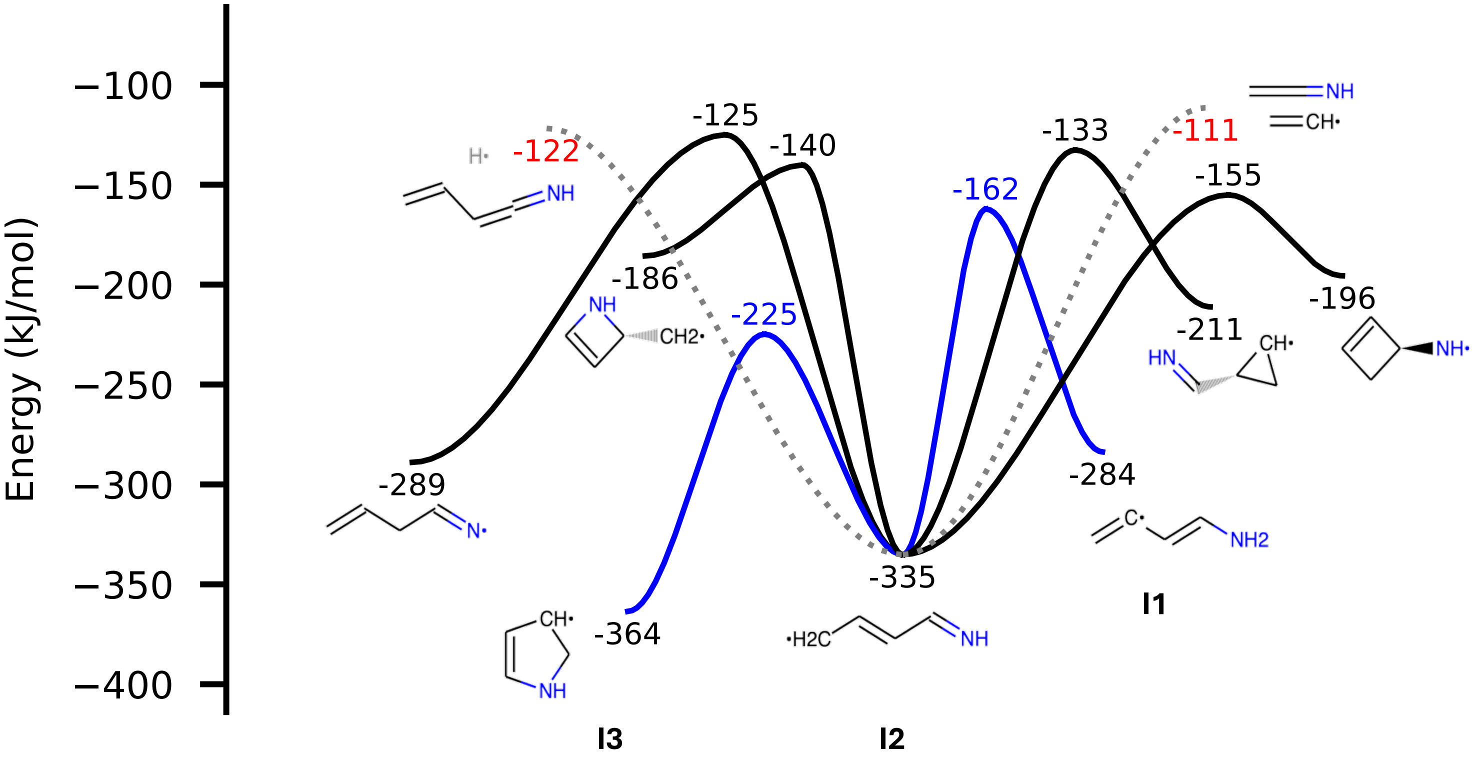

Figure S15: KinBot calculation starting from **I2**, calculated at M06-2X/aug-cc-pVTZ.

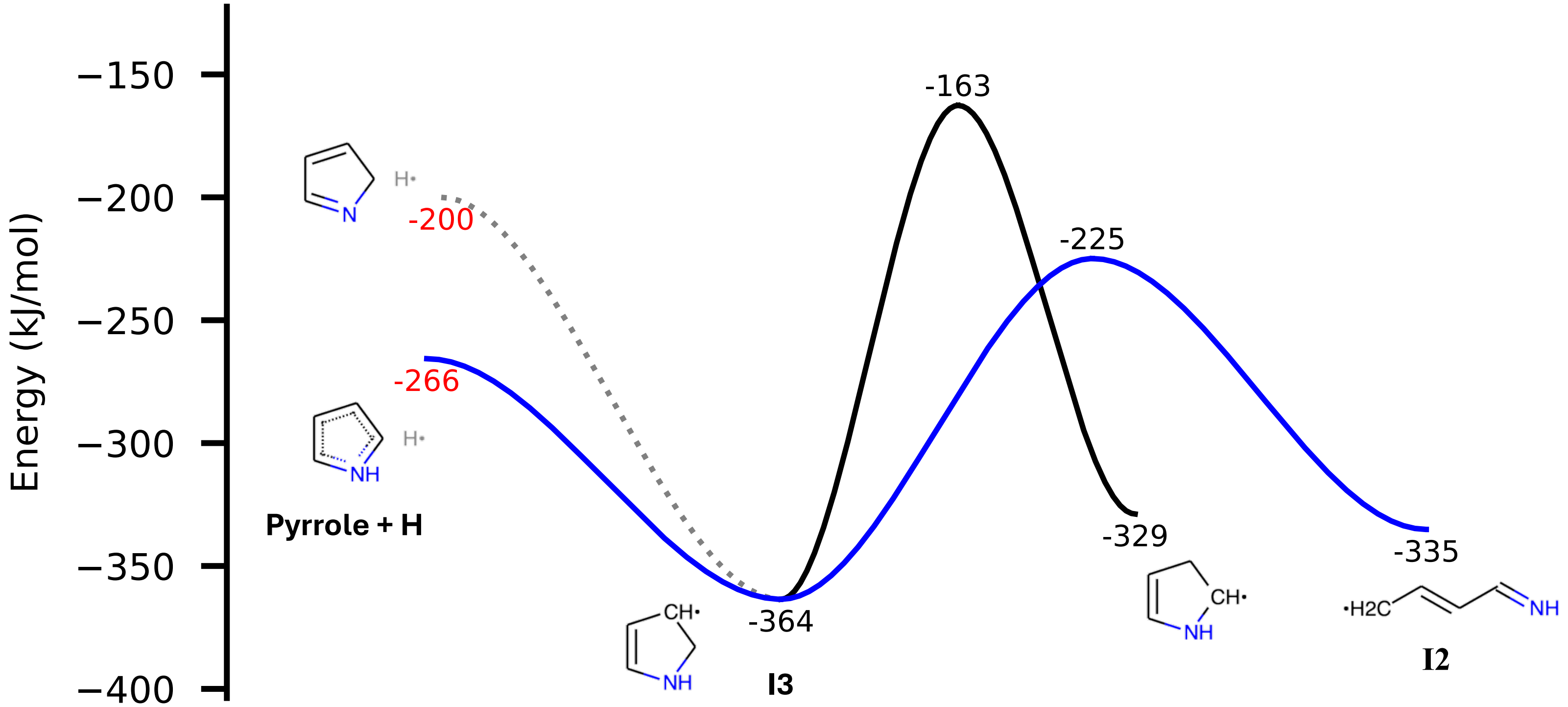

Figure S16: KinBot calculation starting from **I3**, calculated at M06-2X/aug-cc-pVTZ.

are purely from the DFT level, as only the lowest stationary points detailed in the summary PES shown in the main text (Fig. 3) included subsequent single-point calculations at the CCSD(T) level.

Regardless of the specific conformer of intermediates **I1–I3** found across each of the KinBot jobs, we list the lowest conformer found for these intermediates for consistency. Finally, to clarify the pathway followed in the series of KinBot reaction searches, the lowest energy reaction steps connecting the C_4H_6N intermediates **I1–I3** along the **AM-headPR** to pyrrole + H are highlighted in blue.

Fig. S14 shows the reaction search from initial **AM-headPR** intermediate **I1**. In cases where distinct hydrogen migrations lead to the same intermediate, the lower barrier is kept for the plot. Further trimming of pathways cut out hydrogen transfer steps and one ring closure (to a similar intermediate as depected in 151 kJ/mol), all with higher energy barriers such that 9 out of 14 pathways are kept in the plot from the output of the calculation and are plotted in Fig. S14. KinBot successfully identifies the bond-dissociation to reactants **AM** and **PR** referenced at 0 kJ/mol. Furthermore, the reaction path to **I2** is the step with the lowest barrier (**TS1-2** at −162 kJ/mol). KinBot finds that there is another TS at −145 kJ/mol, about 17 kJ/mol above **TS1-2**, leading to the resonance stabilized substituted cyclopropene radical derivative, (S)-2-Methyl-2-cyclopropen-1-ylamine (**I7**). The energy difference between **TS1-2** and **TS1-7** is the lowest energy gap found between the lowest and second lowest energy TS on the entire **AM-headPR** potential energy surface. It thus represents a good case to test how selective the **AM-headPR** mechanism is towards pyrrole formation using statistical kinetics calculations on the computed PES. We first refine the single point energy of both **TS1-2** and **TS1-7** with CCSD(T) and then calculate the forward RRKM microcanonical rate constants as a function of energy starting from **I1** to both **I7** and **I2**. At an internal energy equal to the entrance channel, i.e., the energy of the **AM** and **PR** system at 0 K, the branching ratio is calculated to be 75% to **I2** and, thus, pyrrole formation, whereas 25% to **I7**. The calculated kinetics are shown in Fig. S17, in

which the entrance channel energy is taken as 0 kJ/mol (vertical red line). Additionally, a KinBot calculation on **I7** reveals that the lowest barrier connected to **I7** is back to **I1**, and none of the other higher lying TS connect to intermediates feasible for pyrrole formation. Since **TS1-2** subsequently results in bimolecular products that do not recombine, the reac-

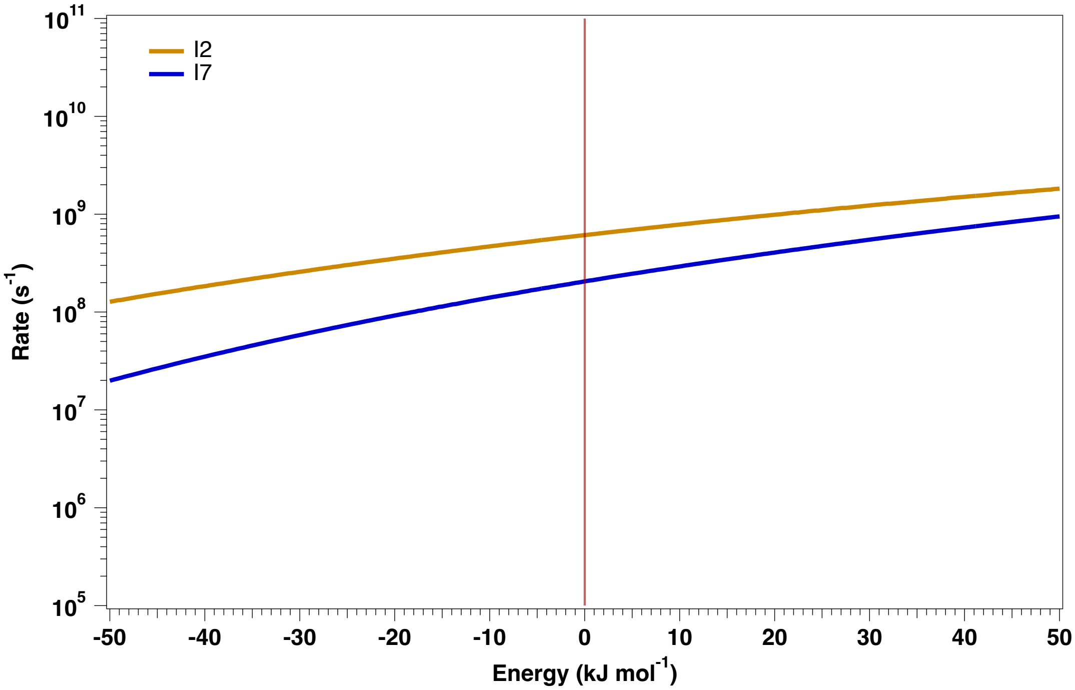

Figure S17: Formation rates of **I2** and **I7** from the initial intermediate formed from reaction of **AM** with the CH$^{\cdot}$ end of **PR**

tion flux would dominantly be expected to proceed through **TS1-2**. Notably, since **TS1-2** is the highest energy TS along the entire **AM-headPR** pathway, as is apparent from the subsequent isomerization steps and TS barriers, it is reasonable to expect that **TS1-2** could be the overall rate-limiting TS for pyrrole formation. Therefore, on the basis that our test of **TS1-2** vs **TS1-7** is a rational estimate how of a minor branching fraction and an expanded view of the nearby portion of the PES afforded by kinbot can predict product formation independent of the experimental results, higher lying TS identified by KinBot are thus considered a dead end for pyrrole formation. The finding that **I2** is the major product starting from **I1** lends credence to the strategy used here of following the lowest energy reaction pathway in each KinBot calculation and indicates that pyrrole is likely the dominant product from the

association of **AM** and **PR**.

The next step in the reaction is shown in Fig. S15, in which **I2** is the input structure. Similar to Fig. S15, a high lying H-loss step and a duplicate hydrogen migration are trimmed for clarity. KinBot finds the path back to **I1** and identifies the lowest barrier step as ring-closure to **I3** via **TS2-3** at -227 kJ/mol relative to the reactants. The only TS that is lower in energy than **TS1-2** is **TS2-3**, and therefore no alternative reaction steps are expected to be competitive with ring closure of **I2** to **I3**.

In the final plot, Fig. S16, the formation of the reaction products pyrrole+H$^{\cdot}$ are found to be lowest in energy. Similar to the case of **I2**, there is only one energetically favorable step below the preceeding TS, indicating that while pyrrole+H$^{\cdot}$ is higher in energy than **I3**, the bimolecular products are expected to form irreversibly and thus consitute the major products. Below a threshold of 220 kJ/mol above **I3**, only select hydrogen migrations across the pyrrolidine ring and two H-loss pathways are accessible. Approximately 71 kJ/mol above pyrrole formation lies the H-loss to 2H-pyrrole, an isomer of pyrrole with a calculated IE of 9.50 eV (Table S2). This pathway is unlikely to be competitive with the formation of pyrrole, and we are not able to verify its presence experimentally. In summary, this investigation provides evidence that the formation of pyrrole+H$^{\cdot}$ is the energetically and kinetically most favorable pathway in the reaction of **AM** with the 'head' of **PR**.

We note that **I3** would not be expected to survive this reaction under either the experimental conditions ($\sim 1000K$, relatively high P) or those of cold molecular clouds ($\sim 10K$ and only $10^4\,\mathrm{cm}^{-3}$). **I3** is relatively weakly bound compared to pyrrole and is formed over a TS (TS2-3) of +140 kJ/mol. This means that **I3** could only be stabilized in a cold *and* high-pressure environment, in which its re-thermalization/stabilization is faster than H-loss to pyrrole +H$^{\cdot}$ (an irreversible step in both the experiment and in the ISM). In the ISM, **I3** would be formed with excess internal energy at least that of the sum of the reactants, about +360 kJ/mol, where relaxation via infrared emission would not be fast enough to stabilize **I3** in its potential well. There, **I3** would lose H$^{\cdot}$ (the H-atom carries away part of the excess

energy) and pyrrole sits in a deep energy well.

3.4 KinBot study of the AM-tailPR Reaction Mechanism

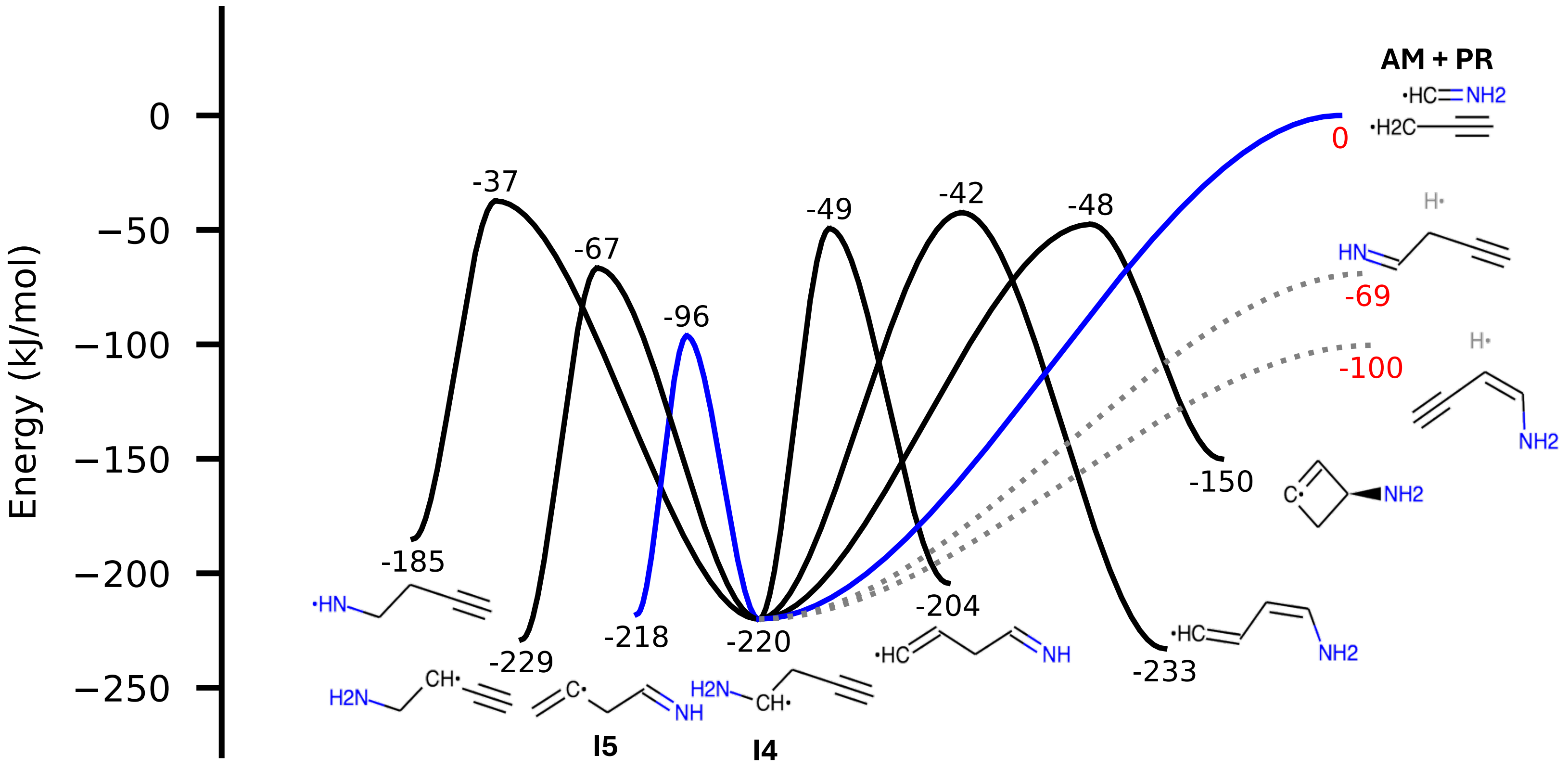

Figure S18: KinBot calculation starting from **I4**, calculated at M06-2X/aug-cc-pVTZ. While at the DFT level the H-loss route found at −100 kJ/mol is lower than the TS at −96 kJ/mol, a subsequent calculation at CCSD(T) finds that the TS sits at −102 kJ/mol above **I4** while the H-loss route is higher at −94 kJ/mol.

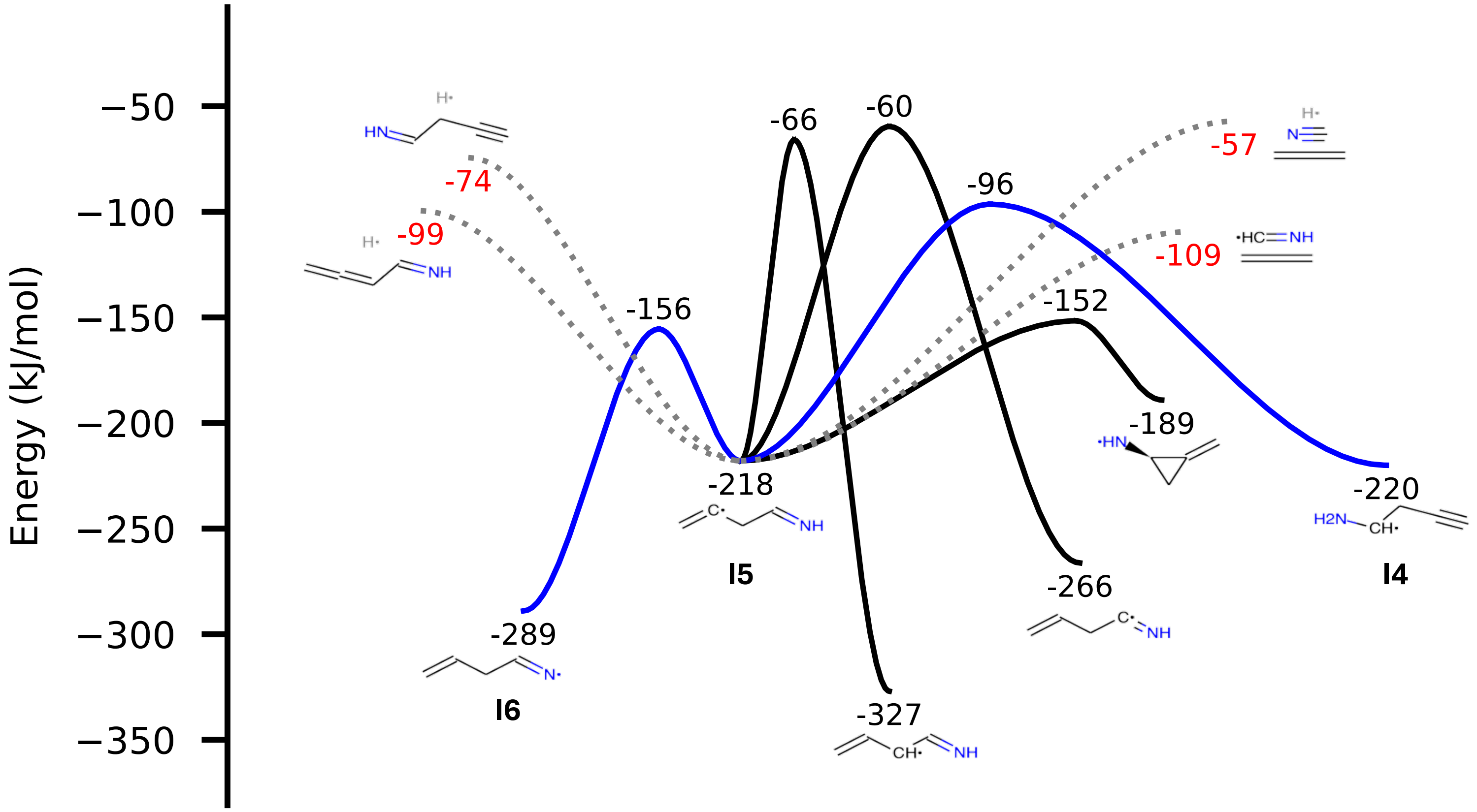

Figure S19: KinBot calculation starting from **I5**, calculated at M06-2X/aug-cc-pVTZ.

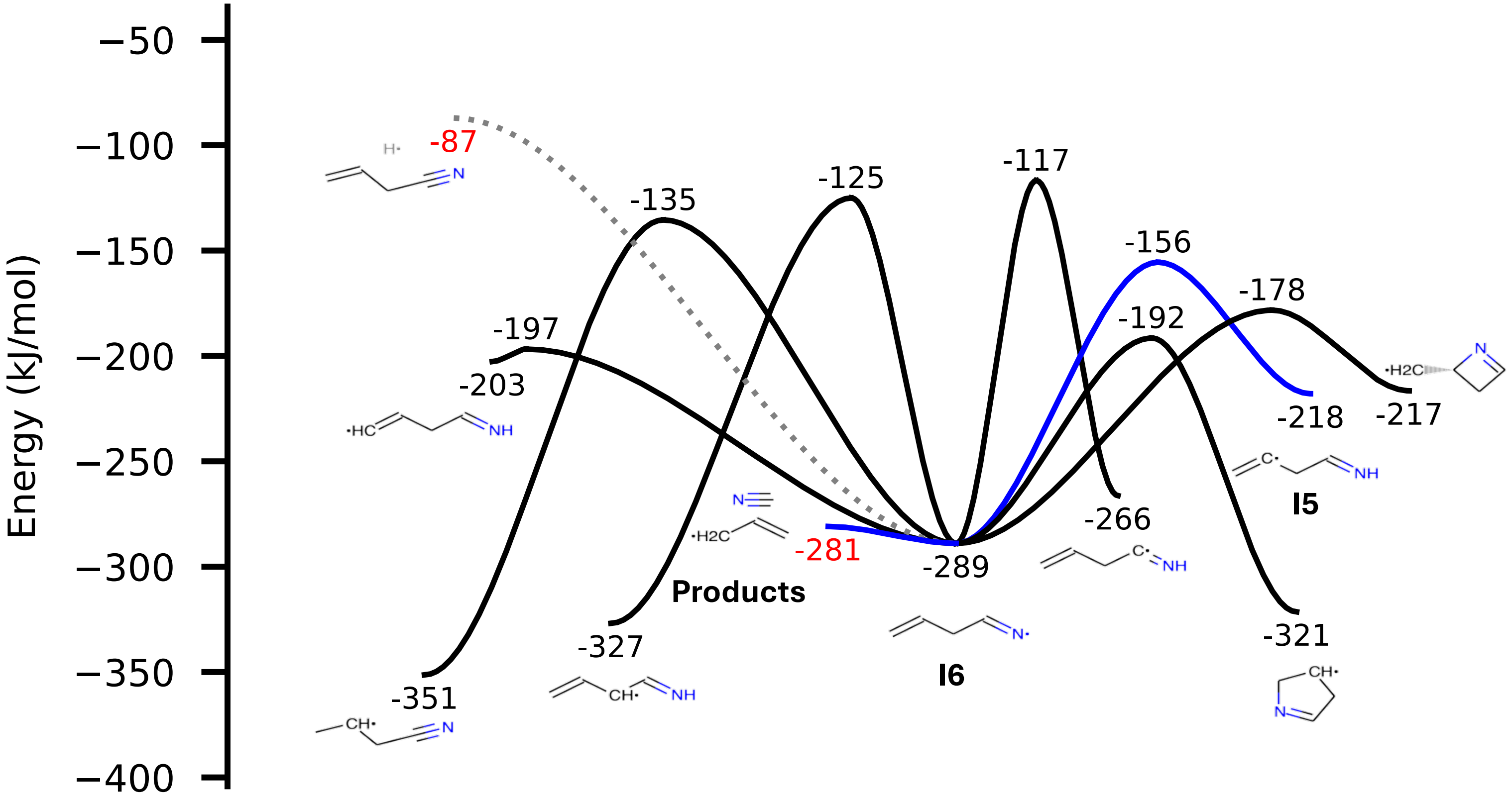

Figure S20: KinBot calculation starting from **I6**, calculated at M06-2X/aug-cc-pVTZ.

3.5 Consideration of other C_4H_5N isomers of m/z 67

The mass spectrometry results reveal ion signal at m/z 67 in the 8.5 eV co-flow mass spectrum. Beyond the characterization of pyrrole as a carrier of the ion peak by ms-TPES, we also consider additional C_4H_5N species as potential reaction products. Several are identified by KinBot as H-loss pathways from various C_4H_6N intermediates. However, based on the calculated energetics (Figs. S14–20), none of the H-loss pathways are expected to be competitive with C_4H_6N unimolecular rearrangement except in the case of pyrrole formation. Any C_4H_5N isomers with IEs above that of pyrrole (8.2 eV) would feasibly be collected in our mass spectrometer when photoionizing with higher photon energies, but their ion signal would overlap with the pyrrole peak in the all-electron PI-TOF-MS. No noticeable increase in m/z 67 ion signal in mass spectra collected with higher photon energies was observed, although attempting to scan to photon energies higher than 8.7 eV and extract a ms-TPES is complicated by increasing contamination from the strong CPA signal lowering the dynamic range of the experiment. Thus, for the high IE C_4H_5N isomers, the experimental results do not concretely comment on their presence, and the evidence for their non-formation is primarily based on the calculated PES.

As seen in Table 2, three C_4H_5N isomers have IEs lower than that of pyrrole. Thus, any ion signal at m/z 67 in the co-flow mass spectrum obtained with a photon energy of 8 eV would be an indication of these isomers. We do not observe any ion peak at m/z 67 in that spectrum. Thus, while our present experimental results do not completely rule out the possibility of additional C_4H_5N isomers beyond pyrrole produced by a reaction with propargyl radicals (particularly in trace amounts), the absence of at least the low IE isomers is consistent with the computational findings that suggest noncompetitive formation of additional C_4H_5N isomers. Table S2 includes the calculated ionization energies for the set of isomers identified in the course of our KinBot study, as well as the C_4H_5N species listed on the NIST Chemistry Webbook. Finally, we note that pyrrole is the most energetically stable isomer in this set of C_4H_5N species.

Table S2: Set of C_4H_5N isomers identified by KinBot and their ionization energies calculated using CBS-QB3 unless otherwise referenced

Structure	IE (eV)	Structure	IE (eV)
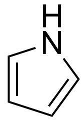	8.20[43]	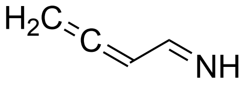	9.40
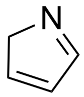	9.50	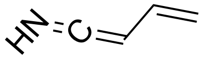	7.85
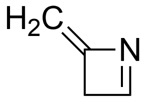	9.49	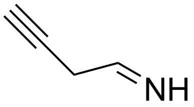	9.65
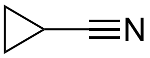	10.25[44]	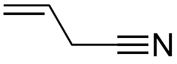	10.22[44]
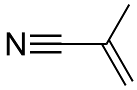	10.34[44]	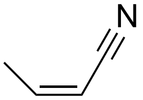	10.23
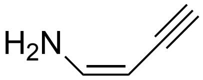	7.86	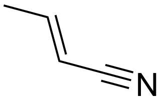	10.23[45]
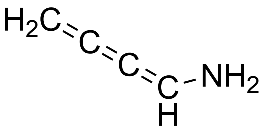	7.51		

References

(1) Bodi, A.; Johnson, M.; Gerber, T.; Gengeliczki, Z.; Sztáray, B.; Baer, T. Imaging photoelectron photoion coincidence spectroscopy with velocity focusing electron optics. *Rev. Sci. Instrum.* **2009**, *80*, 034101.

(2) Johnson, M.; Bodi, A.; Schulz, L.; Gerber, T. Vacuum ultraviolet beamline at the Swiss Light Source for chemical dynamics studies. *Nucl. Instrum. Methods Phys. Res., Sect. A* **2009**, *610*, 597–603.

(3) Bodi, A.; Hemberger, P.; Gerber, T.; Sztáray, B. A new double imaging velocity focusing coincidence experiment: i2PEPICO. *Rev. Sci. Instrum.* **2012**, *83*, 083105.

(4) Tang, W.; Tranter, R. S.; Brezinsky, K. Isomeric Product Distributions from the Self-Reaction of Propargyl Radicals. *J. Phys. Chem. A* **2005**, *109*, 6056–6065.

(5) Guan, Q.; Urness, K. N.; Ormond, T. K.; David, D. E.; Ellison, G. B.; Daily, J. W. The properties of a micro-reactor for the study of the unimolecular decomposition of large molecules. *Int. Rev. Phys. Chem.* **2014**, *33*, 447–487.

(6) Guan, Q.; Ellison, G. B.; Daily, J. W.; Stanton, J. F.; Ahmed, M. DSMC Simulations of a Photoionization Mass Spectrometer. *54th AIAA Aerospace Sciences Meeting* **2016**,

(7) Hemberger, P.; Wu, X.; Pan, Z.; Bodi, A. Continuous Pyrolysis Microreactors: Hot Sources with Little Cooling? New Insights Utilizing Cation Velocity Map Imaging and Threshold Photoelectron Spectroscopy. *J. Phys. Chem. A* **2022**, *126*, 2196–2210, PMID: 35316066.

(8) Sztáray, B.; Baer, T. Suppression of hot electrons in threshold photoelectron photoion coincidence spectroscopy using velocity focusing optics. *Rev. Sci. Instrum.* **2003**, *74*, 3763–3768.

(9) Frisch, M. J. et al. Gaussian 16 Revision C.02. 2016; Gaussian Inc. Wallingford CT.

(10) Van de Vijver, R.; Zádor, J. KinBot: Automated stationary point search on potential energy surfaces. *Comput. Phys. Commun.* **2020**, *248*, 106947.

(11) Zádor, J.; Martí, C.; Van de Vijver, R.; Johansen, S. L.; Yang, Y.; Michelsen, H. A.; Najm, H. N. Automated Reaction Kinetics of Gas-Phase Organic Species over Multiwell Potential Energy Surfaces. *J. Phys. Chem. A* **2023**, *127*, 565–588, PMID: 36607817.

(12) Becke, A. D. A new mixing of Hartree–Fock and local density-functional theories. *J. Chem. Phys.* **1993**, *98*, 1372–1377.

(13) Ditchfield, R.; Hehre, W. J.; Pople, J. A. Self-Consistent Molecular-Orbital Methods. IX. An Extended Gaussian-Type Basis for Molecular-Orbital Studies of Organic Molecules. *J. Chem. Phys.* **1971**, *54*, 724–728.

(14) Dunning, J., Thom H. Gaussian basis sets for use in correlated molecular calculations. I. The atoms boron through neon and hydrogen. *J. Chem. Phys.* **1989**, *90*, 1007–1023.

(15) Kendall, R. A.; Dunning, J., Thom H.; Harrison, R. J. Electron affinities of the first-row atoms revisited. Systematic basis sets and wave functions. *J. Chem. Phys.* **1992**, *96*, 6796–6806.

(16) Van de Vijver, R. PESViewer; 2018. 2023.

(17) Purvis, I., George D.; Bartlett, R. J. A full coupled-cluster singles and doubles model: The inclusion of disconnected triples. *J. Chem. Phys.* **1982**, *76*, 1910–1918.

(18) Sztáray, B.; Bodi, A.; Baer, T. Modeling unimolecular reactions in photoelectron photoion coincidence experiments. *J. Mass Spectrom.* **2010**, *45*, 1233–1245.

(19) Lokachari, N. et al. A comprehensive experimental and kinetic modeling study of di-isobutylene isomers: Part 2. *Combust. Flame* **2023**, *251*, 112547.

(20) Bouchoux, G.; Alcaraz, C.; Dutuit, O.; Nguyen, M. T. Unimolecular Chemistry of the Gaseous Cyclopropylamine Radical Cation. *J. Am. Chem. Soc.* **1998**, *120*, 152–160.

(21) Bouchoux, G.; Gaudin, B.; Leblanc, D.; Yáñez, M.; Mó, O. Is ionized cyclopropylamine cyclic? *Int. J. Mass Spectrom.* **2000**, *199*, 59–69.

(22) Papp, P.; Danko, M.; Štefan Matejčík Electron ionization and photoionization of cyclopropylamine. *Int. J. Mass Spectrom.* **2020**, *455*, 116390.

(23) Luckraft, D. A.; Robinson, P. J. Kinetics of the reactions of cyclopropane derivatives. IV. Kinetic isotope effect in the isomerisation of cyclopropylamine. *Int. J. Chem. Kinet.* **1973**, *5*, 329–331.

(24) Parry, K. A. W.; Robinson, P. J. Kinetics of the reactions of cyclopropane derivatives, part II. The gas-phase pyrolysis of cyclopropylamine. *Int. J. Chem. Kinet.* **1973**, *5*, 27–35.

(25) Hamada, Y.; Amatatsu, Y.; Tsuboi, M. Pyrolysis of amines: Infrared spectrum of 1-aminopropene. *J. Mol. Spectrosc.* **1985**, *110*, 369–378.

(26) Hamada, Y.; Takeo, H. Spectroscopic Study on the Pyrolytic Products of Amines. *Appl. Spectrosc. Rev.* **1992**, *27*, 289–321.

(27) Monascal, Y.; Badenes, M. P. The gas-phase pyrolysis of cyclopropylamine. Quantum chemical characterisation of the intermediates involved. *Molecular Physics* **2021**, *119*, e1814436.

(28) Eckhardt, A. K.; Schreiner, P. R. Spectroscopic Evidence for Aminomethylene (H-C-NH2)-The Simplest Amino Carbene. *Angew. Chem., Int. Ed.* **2018**, *57*, 5248–5252.

(29) Berkowitz, J.; Greene, J. P.; Cho, H.; Ruscić, B. The ionization potentials of CH4 and CD4. *J. Chem. Phys.* **1987**, *86*, 674–676.

(30) Dibeler, V. H.; Liston, S. K. Mass-Spectrometric Study of Photoionization. IX. Hydrogen Cyanide and Acetonitrile. *J. Chem. Phys.* **1968**, *48*, 4765–4768.

(31) Williams, B. A.; Cool, T. A. Two-photon spectroscopy of Rydberg states of jet-cooled C2H4 and C2D4. *J. Chem. Phys.* **1991**, *94*, 6358–6366.

(32) Albrecht, B.; Allan, M.; Haselbach, E.; Neuhaus, L.; Carrupt, P.-A. Molecular Ions of Transient Species: Vinylamine-Cation. *Helvetica Chimica Acta* **1984**, *67*, 220–223.

(33) Gibson, S. T.; Greene, J. P.; Berkowitz, J. Photoionization of the amidogen radical. *J. Chem. Phys.* **1985**, *83*, 4319–4328.

(34) Nesbitt, F. L.; Marston, G.; Stief, L. J.; Wickramaaratchi, M. A.; Tao, W.; Klemm, R. B. Measurement of the photoionization spectra and ionization thresholds of the methyleneamidogen and methyleneamidogen-d2 radicals. *J. Phys. Chem.* **1991**, *95*, 7613–7617.

(35) Gozem, S.; Krylov, A. I. The ezSpectra suite: An easy-to-use toolkit for spectroscopy modeling. *WIREs Comput. Mol. Sci.* **2022**, *12*, e1546.

(36) Holzmeier, F.; Lang, M.; Hader, K.; Hemberger, P.; Fischer, I. H2CN+ and H2CNH+: New insight into the structure and dynamics from mass-selected threshold photoelectron spectra. *J. Chem. Phys.* **2013**, *138*, 214310.

(37) Bock, H.; Dammel, R. Gasphasen-Reaktionen, 60^1) Methanimine RR$'C = NR''$: Darstellung und Photoelektronen-Spektren2–4. *Chemische Berichte* **1987**, *120*, 1961–1970.

(38) Burgers, P.; Holmes, J.; Terlouw, J. Gaseous [H_2, C, N]+ and [H_3, C, N]+. ions. Generation, heat of formation, and dissociation characteristics of [H_2CN]+,[HCNH]+,[CNH_2]+,[H_2CNH]+., and [$HCNH_2$]+.. *J. Am. Chem. Soc.* **1984**, *106*, 2762–2764.

(39) Polce, M. J.; Kim, Y.; Wesdemiotis, C. First experimental characterization of aminocar-

bene. *Int. J. Mass Spectrom. Ion Processes* **1997**, *167-168*, 309–315, In Honour of Chava Lifshitz.

(40) Chai, J.-D.; Head-Gordon, M. Long-range corrected hybrid density functionals with damped atom–atom dispersion corrections. *Phys. Chem. Chem. Phys.* **2008**, *10*, 6615–6620.

(41) Weigend, F.; Ahlrichs, R. Balanced basis sets of split valence, triple zeta valence and quadruple zeta valence quality for H to Rn: Design and assessment of accuracy. *Phys. Chem. Chem. Phys.* **2005**, *7*, 3297–3305.

(42) Grimme, S.; Antony, J.; Ehrlich, S.; Krieg, H. A consistent and accurate ab initio parametrization of density functional dispersion correction (DFT-D) for the 94 elements H-Pu. *J. Chem. Phys.* **2010**, *132*, 154104.

(43) van den Brom, A. J.; Kapelios, M.; Kitsopoulos, T. N.; Nahler, N. H.; Cronin, B.; Ashfold, M. N. R. Photodissociation and photoionization of pyrrole following the multiphoton excitation at 243 and 364.7 nm. *Phys. Chem. Chem. Phys.* **2005**, *7*, 892–899.

(44) Willett, G. D.; Baer, T. Thermochemistry and dissociation dynamics of state-selected C4H4X ions. 3. C4H5N+. *J. Am. Chem. Soc.* **1980**, *102*, 6774–6779.

(45) Houk, K. N.; Munchausen, L. L. Ionization potentials, electron affinities, and reactivities of cyanoalkenes and related electron-deficient alkenes. A frontier molecular orbital treatment of cyanoalkene reactivities in cycloaddition, electrophilic, nucleophilic, and radical reactions. *J. Am. Chem. Soc.* **1976**, *98*, 937–946.
